# Programmable polyketide biosynthesis platform for production of aromatic compounds in yeast

**DOI:** 10.1016/j.synbio.2020.01.004

**Published:** 2020-01-23

**Authors:** Tadas Jakočiūnas, Andreas K. Klitgaard, Eftychia Eva Kontou, Julie Bang Nielsen, Emil Thomsen, David Romero-Suarez, Kai Blin, Christopher J. Petzold, Jennifer W. Gin, Yaojun Tong, Charlotte Held Gotfredsen, Pep Charusanti, Rasmus J.N. Frandsen, Tilmann Weber, Sang Yup Lee, Michael K. Jensen, Jay D. Keasling

**Affiliations:** a The Novo Nordisk Foundation Center for Biosustainability, Technical University of Denmark, Kgs. Lyngby, Denmark; b Joint BioEnergy Institute, Emeryville, CA, USA; c Biological Systems & Engineering Division, Lawrence Berkeley National Laboratory, Berkeley, CA, USA; d Department of Chemical and Biomolecular Engineering & Department of Bioengineering University of California, Berkeley, CA, USA; e Center for Synthetic Biochemistry, Institute for Synthetic Biology, Shenzhen Institutes for Advanced Technologies, Shenzhen, China; f Department of Chemistry, Technical University of Denmark, Kgs. Lyngby, Denmark; g Department of Biotechnology and Biomedicine, Technical University of Denmark, Kgs. Lyngby, Denmark; h Department of Chemical and Biomolecular Engineering (BK21 Plus Program), Korea Advanced Institute of Science and Technology (KAIST), 291 Daehak-ro, Yuseong-gu, Daejeon, 34141, Republic of Korea

**Keywords:** Natural products, Synthetic biology, Aromatic polyketides, CRISPR/Cas9, Polyketide, Production platform in eukaryotes, Metabolic engineering, Biotechnology

## Abstract

To accelerate the shift to bio-based production and overcome complicated functional implementation of natural and artificial biosynthetic pathways to industry relevant organisms, development of new, versatile, bio-based production platforms is required. Here we present a novel yeast-based platform for biosynthesis of bacterial aromatic polyketides. The platform is based on a synthetic polyketide synthase system enabling a first demonstration of bacterial aromatic polyketide biosynthesis in a eukaryotic host.

## 1 Introduction

An increasing number of chemicals are being produced by environmentally-friendly bio-based synthesis [1,2] to overcome the problems of low-yielding chemical synthesis or solvent-heavy extraction from natural resources, for achieving a sustainable way of life. Unfortunately, development of microbial cell factories for the bio-based production of desired chemicals often requires a significant amount of time, resources and efforts to meet industrial demand, hence the shift towards bio-based production is slow. Also in many cases native hosts are not suitable for industrial conditions due to low production level and/or complicated culturing conditions, necessitating the use of a heterologous hosts such as *Escherichia coli* or yeast [3,4]. However, production of natural products, such as polyketides, in heterologous hosts has often proven difficult or even impossible [[5], [6], [7]]. To overcome these limitations, standardized, versatile and programmable biosynthesis platforms in genetically tractable and robust hosts are desired.

Polyketides are a large class of bioactive natural compounds found widely in fungi (type I iterative and type II), bacteria (type I, type II and type III) and plants (type III), possessing a variety of biological activities, including antibacterial, anticancer, antifungal, antiviral and more [[8], [9], [10], [11]]. As a consequence, polyketides have been, and still are, major leads in drug discovery programs [[11], [12], [13], [14]].

The most diverse and widely studied polyketides originate from bacteria [11]. Complex bacterial aromatic polyketides can be produced through non-reducing polyketide pathways, where two-carbon units (-CH2-CO-, ketides) are polymerized into linear polyketide chains of various lengths by multicomponent enzyme complexes known as polyketide synthase (minimal PKS), and then can be folded to form aromatic structures [[15], [16], [17]]. Folding in most bacterial systems is facilitated by aromatases, ketoreductases and cyclases, and the resulting products are further modified by other classes of tailoring enzymes that closely interact with the minimal PKS [18]. From the bioengineering point of view, bacterial type II PKS systems offer flexibility in terms of choice from vast amount of aromatases, cyclases and tailoring enzymes to allow for the rational engineering of pathways to form desirable aromatic compounds [19] and to develop programmable polyketide production platforms [20]. Development and optimization of such a production platform in native bacterial hosts can be troublesome due to the lack of genetic tools, production of unwanted toxic metabolites, undetermined culturing conditions, and low or conditional production of desired compounds [3,4,21]. Unfortunately, bacterial type II PKSs have not yet proven possible to express in eukaryotes [15]. In contrast, plant (type III) PKSs, that ultimately form aromatic compounds via linear non-reduced polyketide intermediates, consist of a single enzyme [22] and can be expressed in heterologous eukaryotic hosts to form a polyketide chain of varied length, yet the lack of characterized cyclases, aromatases and tailoring enzymes in plant PKS systems limits the use of type III PKSs for versatile polyketide biosynthesis [23,24]. Although, recently it was demonstrated that it is possible to functionally combine the activity of plant type III PKS with bacterial type II PKS related cyclase and aromatase in plants and filamentous fungi [24,25].

Here, we describe a first-of-its-kind programmable polyketide production platform in the yeast *Saccharomyces cerevisiae*, based on combining the synthesis of a polyketide (octaketide) by plant-based type III octaketide synthase (OKS) from *Aloe arborescens* to produce type II polyketide products from benzoisochromanequinone antibiotic - actinorhodin pathway (Act) from *Streptomyces coelicolor*.

## 2 Materials and methods

### 2.1 Strains, plasmids and media

The yeast strains used here were isogenic to CEN.PK2–1C. Strains and plasmids are listed in Supplementary Tables 2 and 3, respectively. Yeast cells were grown in complete medium (YPD) with 2% glucose and synthetic complete (SC) from Sigma, supplemented with 2% glucose. *E. coli* strains were propagated in LB medium supplemented with 200 mg of ampicillin, Streptomyces strain was grown in ISP2 medium with 4% glucose.

All primer names and sequences are listed in Supplementary Table 4.

### 2.2 Plasmid and strain construction

To create polyketide expressing strains large set of genes were integrated by using advanced CRISPR/Cas9 technology [26,27]. To create expression cassettes, respective genes were codon optimized for yeast and ordered (Integrated DNA Technologies) as gene blocks. Gene blocks were amplified using corresponding primers (Supplementary Table 4) and first USER cloned with single or bi-directional promoters to yeast integrative plasmids as described in previously published method [26]. All created integrative plasmids with corresponding expression units are listed in Supplementary Table 3. By employing previously detailed procedure [26], all integrative plasmids were linearized and with their corresponding gRNA plasmids transformed into yeast expressing Cas9 for integration of desired genes to the genome. Due to large number of genes to be integrated, this has been processed in 2–3 steps. In a single transformation 4–6 genes were introduced and created strain used for the next round of transformation until all pathway genes were integrated. All the other plasmids and strains were created in the same way as previously described [26].

### 2.3 PKS cluster determination with antiSMASH

In order to collect oktaketide PKS type II clusters, all NBCI assembly IDs for genomes predicted to contain PKS type II clusters were retrieved from the antiSMASH database version 2 [28]. Assemblies indicated by the assembly IDs were downloaded using ncbi-genome-download (https://github.com/kblin/ncbi-genome-download). Next antiSMASH 5.0 [29] was used to reannotate the genomes to make use of the PKS type II chain length predictions [30]. Chain length predictions were collected from the antiSMASH results and filtered for the 8/9 ketide unit extension size.

### 2.4 Metabolite extraction

Yeast cells were cultured in 50–2000 mL SC selective or YPD medium for 168 h at 30 °C, shaking 250 rpm. *Streptomyces* cells were grown in ISP2 medium for 168 h at 30 °C, shaking 250 rpm. Cells were then collected by centrifugation and resuspended in ethyl acetate, which was acidified with 10% acetic acid. Resuspended cells where mixed with 0.5 μM acid washed glass beads (Sigma) and bead beaten to break the cells. Lysates were centrifuged and upper ethyl acetate layer with extracted metabolites was collected. Ethyl acetate was evaporated and extracts resuspended in methanol for further analysis by LC-MS.

### 2.5 Whole cell proteomics

The yeast cultures were grown in YPD medium in triplicates. Exponentially growing cells were harvested (totally OD_600_ - 20) and cell pellets flash-frozen in liquid nitrogen.

To prepare for protein lysis and precipitation, the yeast cell pellets were treated with 0.5 μL (2.5 U) of Zymolyase in 200 μL of 1 M Sorbitol 0.1 M EDTA at 37 °C for 30 min to digest cell walls and centrifuged at 20,817×*g* for 1 min. The supernatant was removed before continuing with a chloroform-methanol extraction as described previously, which was achieved by the addition of 80 μL of methanol, 20 μL of chloroform, and 60 μL of water, with vortexing followed by centrifugation at 20,817×*g* for 1 min to induce phase separation. The methanol and water layer was removed and then 100 μL of methanol was added and the sample with vortexing briefly followed by centrifugation for 1 min. The chloroform and methanol mixture was removed by pipetting to isolate the protein pellet. The protein pellet was resuspended in 100 mM ammonium bicarbonate (AMBIC) with 20% methanol and quantified by the Lowry method (Bio-Rad DC assay). A total of 100 μg of protein was reduced by adding tris(2-carboxyethyl)phosphine (TCEP) to a final concentration of 5 mM for 30 min, followed by alkylation by adding iodoacetamide at a final concentration of 10 mM with incubation for 30 min, and subsequently digested overnight at 37 °C with trypsin at a ratio of 1:50 (w/w) trypsin:total protein.

Peptides were analysed using an Agilent 1290 liquid chromatography system coupled to an Agilent 6460QQQ mass spectrometer (Agilent Technologies, Santa Clara, CA) operating in MRM mode. Peptide samples (10 μg) were separated on an Ascentis Express Peptide ES-C18 column (2.7 μm particle size, 160 Å pore size, 50 mm length x 2.1 mm i.d., 60 °C; Sigma-Aldrich, St. Louis, MO) by using a chromatographic gradient (400 μL/min flow rate) with an initial condition of 95% Buffer A (99.9% water, 0.1% formic acid) and 5% Buffer B (99.9% acetonitrile, 0.1% formic acid) then increasing linearly to 65% Buffer A/35% Buffer B over 5.5 min. Buffer B was then increased to 80% over 0.3 min and held at 80% for 2 min followed by ramping back down to 5% Buffer B over 0.5 min where it was held for 1.5 min to re-equilibrate the column for the next sample. The data were acquired using Agilent MassHunter, version B.08.02, processed using Skyline version 4.1, and peak quantification was refined with mProphet in Skyline. All data and skyline files are available via the Panorama Public repository at this link: https://panoramaweb.org/a-platform-for-polyketide-biosynthesis-using-yeast.url. Data are also available via ProteomeXchange with identifier: PXD013388.

### 2.6 Comparative metabolite profiling by LC-MS and data analysis

LC-MS analysis was performed using a Dionex Ultimate 3000 ultra-high-performance liquid chromatography (UHPLC) coupled to a UV/Vis diode array detector (DAD) and a high-resolution mass spectrometer (HRMS) Orbitrap Fusion mass spectrometer (ThermoFisher Scientific, Waltham, MA, USA). UV–Vis detection was done using a DAD-3000 in the range 200–700 nm. Injections of 5 μL of each sample were separated using a Zorbax Eclipse Plus C-18 column (2.1 × 100 mm, 1.8 μm) (Agilent, Santa Clara, CA, USA) at a flow rate of 0.35 mL/min, and a temperature of 35.0 °C. Mobile phases A and B were 0.1% formic acid in water and acetonitrile, respectively. Elution was performed with a 17 min multistep system. After 5% B for 0.3 min, a linear gradient started from 5% B to 100% B in 13 min, which was held for another 2 min and followed by re-equilibration to 5% B until 17 min. HRMS was performed in ESI-, with a spray voltage of 2750 V respectively, in the mass range (*m/z*) 100–1000 at a resolution of 120,000, RF Lens 50%, and AGC target 2e5. Before analysis, the MS was calibrated using ESI Negative on Calibration Solution (P/N 88324, Thermo Scientific, San Jose, USA).

LC-MS/MS analysis was carried out using data-dependent MS/MS analysis by analyzing the most intense ions form the full-scan using a master scan time of 1.0 s. Dynamic exclusion was used to exclude ions for 20 s after two measurements within 30 s. Fragmentation was performed using stepped HCD collision energy of 15, 25, and 35% at a resolution of 30,000, RF Lens 50%, and AGC target 1e5, while full-scan resolution was set to 60,000.

Data analyses were performed with the software Xcalibur 3.1.2412.17 (Thermo Fisher Scientific Inc.).

### 2.7 Isolation of DMAC

The compound isolation was performed in three steps. The crude extract was initially dissolved in 3.5 mL of 3:1 acetonitrile:methanol and separated using a Dionex Ultimate 3000 HPLC coupled to a UV/Vis diode array detector (DAD) operated under the following conditions: column, Waters XBridge BEH Amide OBD Prep, 130 Å, 5 μm, 10 × 250 mm; column temperature, 30 °C; solvent A, (CH_3_CN) and solvent B (H_2_O buffered with 10 mM (NH_4_)_2_CO_3_, pH 6.5). The mobile phase was: isocratic 0–2 min at 95% A; gradient 2–20 min from 95 to 60% A; isocratic 21–26 min at 10% A. The column was equilibrated for 18 min prior to each injection. The flow rate was 3.0 mL/min. A peak that eluted at 5.2 min with UV λ_max_ of 390 nm was manually collected. The fraction was dried using a rotary evaporator and redissolved in 50% v/v MeOH:water to a final volume of 800 μL. The sample was then subjected to a second, reverse-phase separation under the following conditions: column, Agilent ZORBAX Eclipse Plus Phenyl-Hexyl 95 Å 3.5 μm, 4.6 × 150 mm; column temperature, 30 °C; solvent A (CH_3_OH) and solvent B (H_2_O buffered with 10 mM NH_4_HCO_2_, pH 3.0); step gradient profile: 0–8 min, 50% A; 9–15 min, 60% A; 16–22 min, 70% A; 23–28 min, 95% A; 29–35 min, 50% A; flow rate, 1.5 mL/min. A peak that eluted at 12.2 min with UV λ_max_ of 226, 278 and 390 nm was manually collected. After drying the fraction and redissolving it again in 50% v/v MeOH/water, it was subjected to a third reverse-phase separation under the following conditions: column, Waters XBridge C18 3.5 μm, 4.6 × 150 mm; column temperature, 30 °C; solvent A (CH_3_OH) and solvent B (H_2_O buffered with 10 mM (NH_4_)_2_CΟ_3_, pH 6.5); step gradient profile: 0–8 min, 50% A; 9–15 min, 60% A; 16–22 min, 70% A; 23–28 min, 95% A; 29–35 min, 50% A; flow rate, 1.5 mL/min. The peak of interest eluted at 3.2 min and was manually collected. The sample was dried using a rotary evaporator and most of it redissolved in DMSO-d6 for NMR analysis. A small portion of the sample was dissolved in 50% v/v MeOH/water for MS and MS/MS analysis using the UHPLC system and Orbitrap HRMS.

### 2.8 NMR data acquisition and analyses

NMR spectra were recorded in DMSO-*d6.* All NMR spectra were acquired at 25 °C on a Bruker Avance III 800 MHz spectrometer equipped with a TCI Cryoprobe. All spectra 1D ^1^H, 2D DQF-COSY, HSQC, HMBC, H2BC were acquired using standard pulse sequences. All spectra were processed using TOPSPIN 3.6.1 software (Bruker).

## 3 Results

### 3.1 Characterisation of polyketide gene clusters

**Fig. 1:**
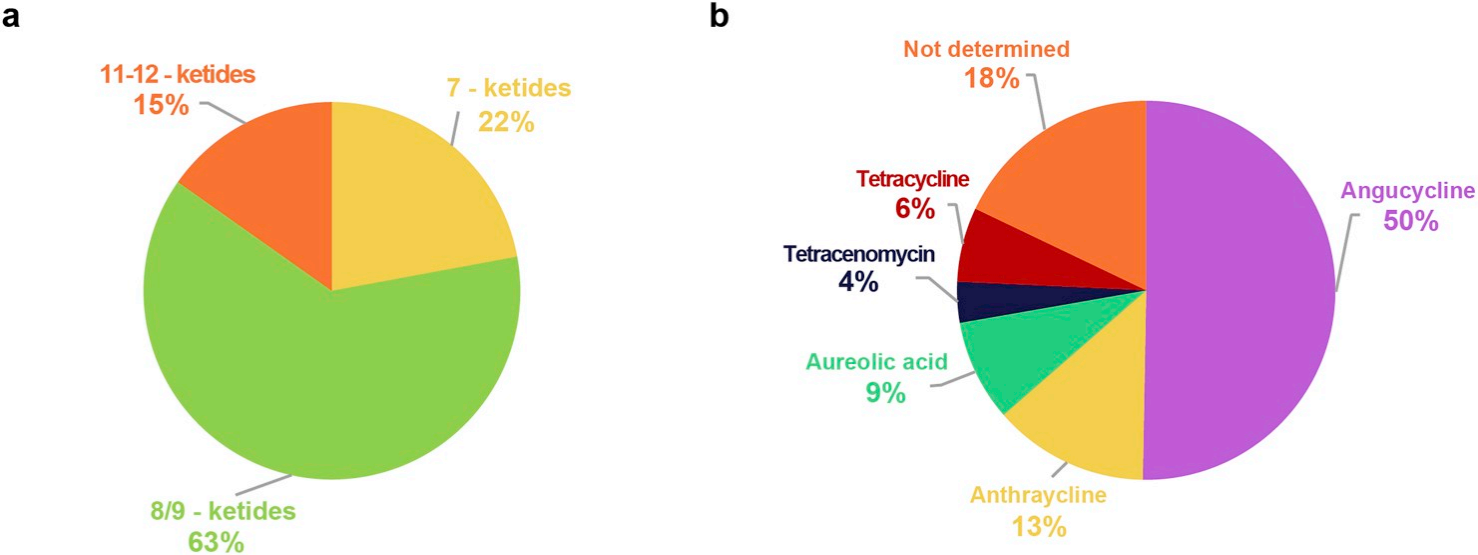
**Characterisation of polyketide gene clusters.** (A) Prediction of the number of ketide extensions units in type II polyketides, majority (63%) of them being 8/9 ketides. (B) Elucidation of functional classes to which the predicted 8/9 ketides belong to.

Prior to choosing and building a gene cluster in yeast, we aimed to determine the number and composition of type II bacterial polyketide gene clusters. We aimed to do this by looking at the sequence data available in the databases and employing antiSMASH v.5 [28]. By set parameters (see Materials and methods) we were able to predict approximately 600 type II polyketide gene clusters. For approximately half of the gene clusters (270) we were able to predict the number of ketide extensions, majority (63%) of them being 8/9 ketides (Fig. 1A). We subsequently aimed to predict the functional classes to which they belong (Fig. 1B). From the computational analysis we determined that 50% of the predicted 8/9 ketides belong to angucycline functional class, followed by 13% anthracycline, 9% aureolic acid, 6% tetracycline and 4% tetracenomycin. Due to lack of annotation information we were not able to predict functional classes for 18% of the 8/9 ketides. Since, the 8/9 ketides were the most abundant type II polyketides among sequenced bacteria, we further decided to concentrate on producing octaketides in yeast.

### 3.2 Expression of actinorhodin biosynthesis pathway in yeast

**Fig. 2:**
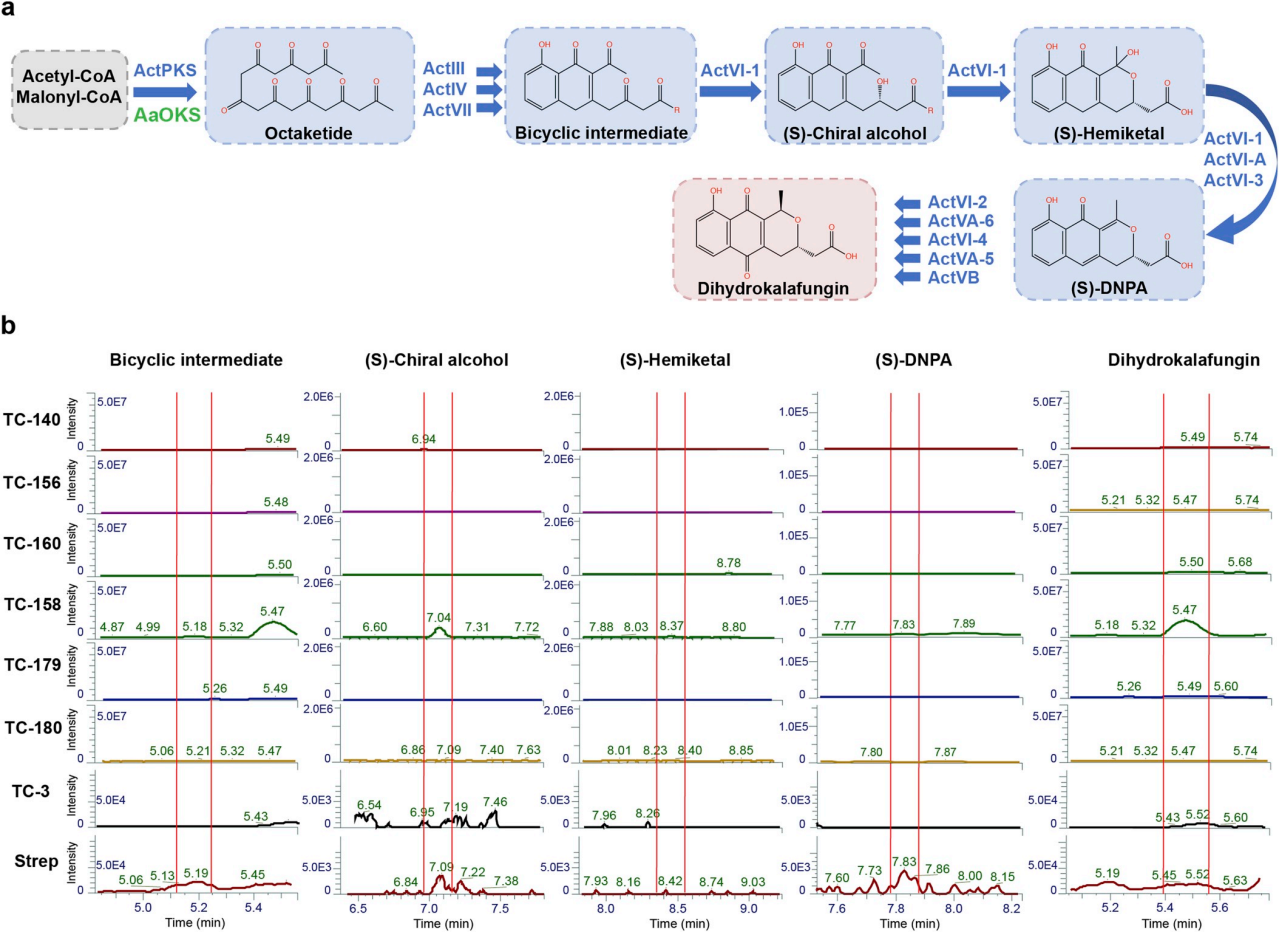
**Expression of actinorhodin biosynthesis pathway in yeast and replacement of Act minimal PKS with *Aa*OKS.** (A) Schematic overview of actinorhodin pathway with Act minimal PKS or with *Aa*OKS expressed in yeast, including enzymes and chemical compounds produced through the pathway. Enzymes are listed in blue and green, blue arrows depict chemical reactions catalysed by listed enzymes and produced compounds are depicted in blue or red shade. (B) Chromatograms from comparative LC-MS metabolomics showing investigated metabolites in yeast. The main products or intermediates investigated by LC-MS were bicyclic intermediate, (S)-chiral alcohol, (S)-hemiketal, (S)-DNPA, dihydrokalafungin (DHK). Metabolites from the natural actinorhodin producer *S. coelicolor* (abbreviated Strep) were used as a standard (positive control) for comparing metabolites produced in *wt* and engineered yeast strains: TC-3, TC-140, TC-160, TC-156, TC-158, TC-179, TC-180. Red vertical lines on chromatograms depict the peaks of listed compounds based on known mass and positive control. Intensities of the peaks and elution times are shown on the corresponding axis.

First, as a proof-of-concept, we reconstructed a pathway of the widely studied and most known octaketide-derived actinorhodin from *S. coelicolor* (NCBI GenBank: AL645882.2) [31,32] by integrating required codon optimized genes (See codon optimized sequences in Supplementary info) into the yeast genome together with its bacterial minimal PKS (Fig. 2A). Gene assemblies and genomic integrations were performed in 2–3 steps by first performing *in vivo* assembly of expression units in *Escherichia coli,* and second by using our recently developed CRISPR/Cas9 genome engineering techniques to integrate the assembled gene expression units into the yeast genome (Supplementary Table 1) [26,27]. Since actinorhodin and other intermediates in the pathway have color [33], successful production through the pathway was initially expected to be assessed by visual inspection of engineered yeast. However, from the first designs, no apparent or very modest color was observed in yeast cells harboring actinorhodin pathway (Supplementary Fig. 1; TC-140, TC-156). To mitigate the lack of (or modest) visual phenotypes we next investigated if all proteins from the Act pathway were successfully expressed using whole cell proteomics. From this analysis it was evident that most of Act proteins were detected except for ActVI-2 (dehydrogenase) and ActI-1 (3-oxoacyl-ACP synthase), the later of which is needed for the first committed step of the minimal ActPKS (Supplementary Fig. 2). Further, we aimed to elucidate which metabolites, if any, are produced from first generation Act pathway design. Since none of the reported pathway metabolites are commercially available as analytical standards, we performed comparative LC-MS metabolite profiling using wild type *S. coelicolor* whole cell extract as a standard. This analysis indicated that none of the described intermediates from the Act pathway were detected in the engineered yeast strains (Fig. 2B; TC-140, TC-156), hinting that the Act - type II PKS indeed was not functionally expressed or correctly assembled into a functional PKS in yeast.

### 3.3 Replacement of Act minimal PKS with *Aa*OKS

To overcome the lack of function of type II PKS we replaced the Act minimal PKS with a type III octaketide synthase from plant *A. arborescens* (*Aa*OKS) [34], which was described to produce a polyketide product with an identical chain-length as Act. Hence, the second generation of yeast production strains were created by replacing the actinorhodin minimal PKS with *AaOKS*, but retaining the rest of the actinorhodin pathway (Fig. 2A). The first strain design expressing *AaOKS* indeed displayed pigmented colonies indicating the production of Act cluster metabolites (Supplementary Fig. 1; TC-158). We further investigated expression optimization of *AaOKS* by integrating either a single-copy of the *AaOKS* gene into the genome or expressing the *AaOKS* from a high-copy plasmid. As judged from phenotypic inspection, only the Act pathway strain expressing *AaOKS* from a high-copy plasmid gave rise to pigmented colonies potentially derived from Act pathway metabolites (Supplementary Fig. 1; TC-160 vs. TC-158). Further, from comparative metabolite profiling, the four main products (bicyclic intermediate, (S)-chiral alcohol, (S)-hemiketal, dihydrokalafungin) from the Act pathway were tentatively observed in this strain (Fig. 2B; TC-158), while no production was observed in the yeast *wt* control (TC-3). In addition, no production was also observed in control strains i) expressing Act pathway without a PKS (TC-156), ii) expressing Act pathway and single-copy of *AaOKS* (TC-160), iii) yeast *wt* control expressing *AaOKS* in either single- or high-copy (TC-179 or TC-180) without the Act pathway (Fig. 2B). The other major intermediate (S)-DNPA from the Act pathway could not be detected or confirmed reliably, most likely because this product was metabolized rapidly by Act pathway enzymes. By comparative LC-MS analyses, based on known mass and published UV data [35], and direct comparison to *S. coelicolor* metabolites, we observed accumulation of a compound expected to be dihydrokalafungin (DHK), as a final product (Fig. 2B; TC-158). To further investigate the compound putatively identified as DHK, we performed more thorough analysis including LC-MS/MS, as no analytical standards were commercially available. These results indicated that DHK was indeed being produced in the engineered yeast strain as its MS/MS fragmentation pattern was the same in both engineered yeast strain and *S. coelicolor* (Supplementary Fig. 3).

### 3.4 Optimization of aromatic polyketide production platform

**Fig. 3:**
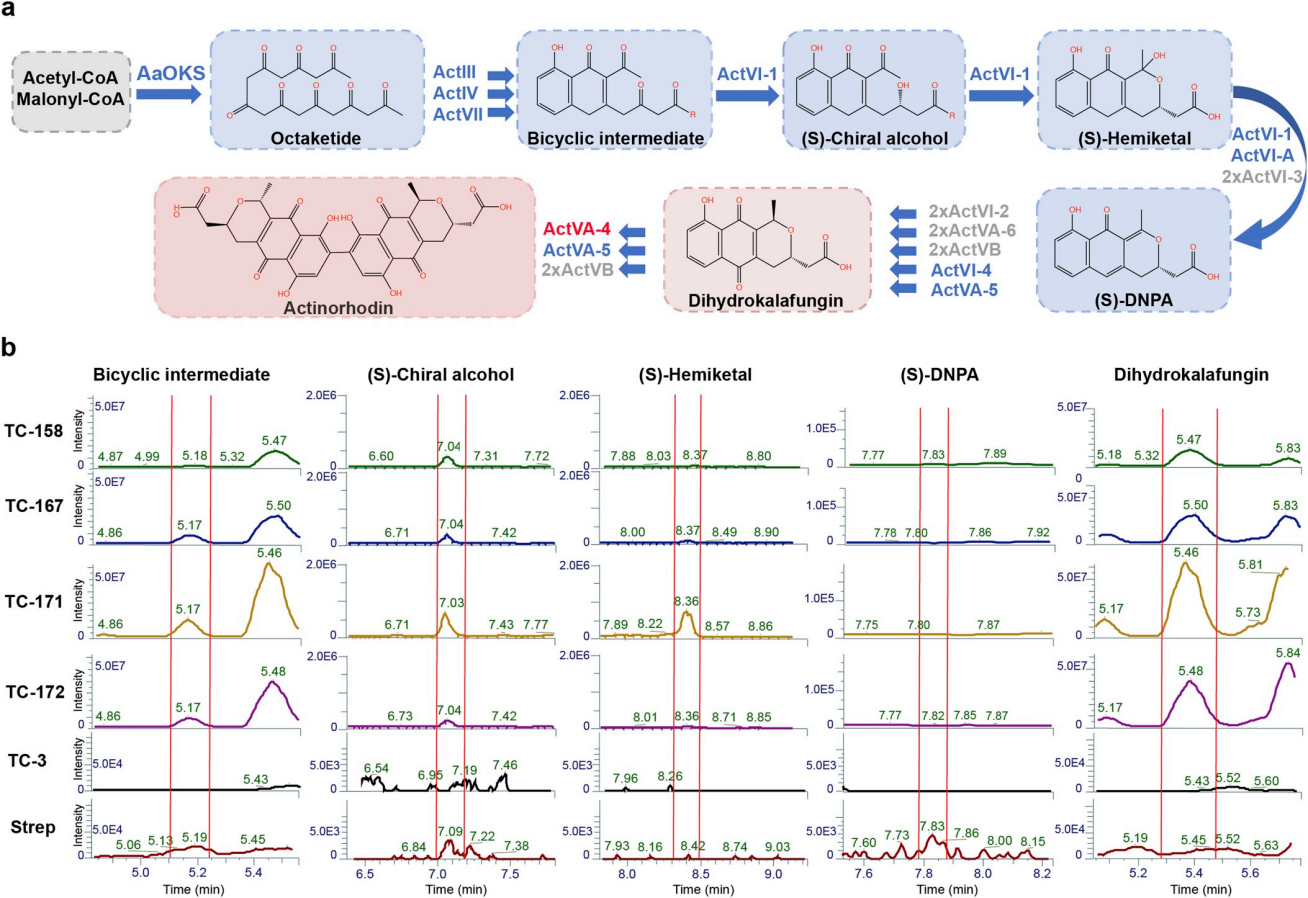
**Optimization of aromatic polyketide production platform.** (A) Schematic overview of the optimized actinorhodin pathway where the Act minimal PKS is replaced with *Aa*OKS in yeast, including enzymes and chemical compounds produced through the pathway. Enzymes encoded by a single-copy gene are indicated in blue, and enzymes encoded by multi-copy genes are indicated in grey. Blue arrows depict chemical reactions catalysed by listed enzymes and produced compounds are depicted in blue or red shade. Dimerize to form actinorhodin is shown in red. (B) Chromatograms from comparative LC-MS metabolomics showing investigated metabolites in optimized yeast strains. Main products or intermediates were investigated by LC-MS: bicyclic intermediate, (S)-chiral alcohol, (S)-hemiketal, (S)-DNPA, dihydrokalafungin (DHK). Metabolites from natural actinorhodin producer *S. coelicolor* (abbreviated Strep) was used as a standard (positive control) for comparing metabolites produced in *wt* and engineered yeast strains: TC-3, TC-158, TC-167, TC-171, TC-172. Red vertical lines on chromatograms depict the peaks of listed compounds based on known mass and positive control. Intensities of the peaks and elution times are shown on the corresponding axis.

To optimize the platform strain further for production of type II polyketide compounds, we next integrated a second copy of each of the four genes encoding ActVI-3, ActVI-2, ActVA-6, ActVB (Fig. 3A), all showing low abundances as evaluated from whole cell proteomics analysis (Supplementary Fig. 4). Upon overexpression of the four genes, the colonies became more intensely colored (Supplementary Fig. 1; TC-167). To investigate if this phenotype could be correlated with increased DHK production, we performed comparative metabolite profiling by LC-MS, and noted that production of the DHK was relatively increased (Fig. 3B; TC-158 vs. TC-167).

Next, we aimed to produce one of the most widely studied model antibiotics, actinorhodin, (a dimer of DHK) in both non-optimized (TC-158) and optimized (TC-167) yeast platform strains. DHK dimerisation, as previously described [35], is potentially catalysed by the enzyme ActVA-4 (Fig. 3A), which initially was not introduced into the yeast platform strains. Introduction of the dimerase should allow for dimerisation of DHK and production of actinorhodin. However, as judged by the color of yeast colonies (Supplementary Fig. 1; TC-171 with the non-optimized Act pathway and TC-172 with the optimized Act pathway), no significant changes were observed after the introduction of dimerase. Further analysis by comparative LC-MS corroborated the phenotypic result revealing no detectable actinorhodin in the yeast strains (Supplementary Fig. 5). In addition, actinorhodin and its intermediates can also be toxic to yeast, and accumulated amounts could inhibit cell growth, and potentially compromise actinorhodin production. We tested this hypothesis by growing *wt* yeast in serial dilutions of conditioned medium where *S. coelicolor* had previously been grown. According to LC-MS analysis (Fig. 2B), this medium contained Act pathway metabolites together with actinorhodin. Here it was observed that growth of yeast cells was completely inhibited in 2x and 4x diluted conditioned medium, and even modestly compromised in 8x diluted conditioned medium (Supplementary Fig. 6), while yeast cultivated in standard ISP2 medium displayed normal growth behaviour, thus indicating toxicity of *S. coelicolor* derived metabolites. Further, it was also observed that engineered yeast strains, producing DHK, showed reduced growth, as judged by colony size and growth profiling (Supplementary Fig. 1; strains: TC-158, TC-167, TC-171; Supplementary Fig. 7).

### 3.5 Programmability of the system

**Fig. 4:**
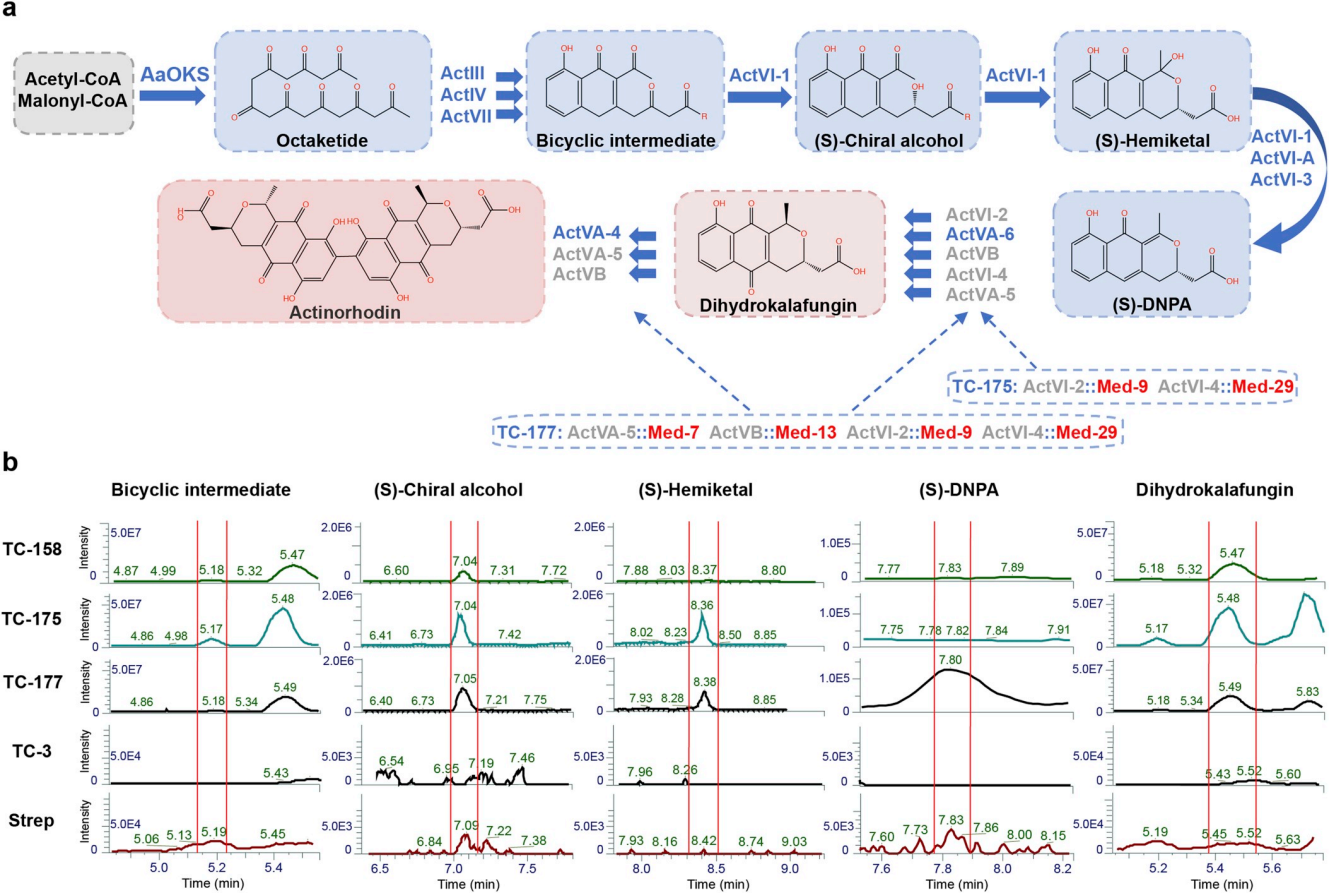
**Programmability of the developed polyketide expression platform.** (A) Schematic overview of the re-programmed actinorhodin pathway where ActVI-2, ActVI-4, ActVA-5, ActVB (native Act pathway enzymes) were functionally replaced with enzymes Med-9, Med-29, Med-7, Med-13 respectively (from medermycin biosynthetic pathway) to test the programmability of the heterologous pathway. Enzymes are listed in blue and Act pathway enzymes which were replaced in grey; enzymes which were newly integrated in to yeast genome shown in red. Blue arrows depict chemical reactions catalysed by listed enzymes and produced compounds are depicted in blue or red shade. (B) Chromatograms from comparative LC-MS metabolomics showing investigated metabolites in reprogrammed yeast strains. Main products or intermediates were investigated by LC-MS: bicyclic intermediate, (S)-chiral alcohol, (S)-hemiketal, (S)-DNPA, dihydrokalafungin (DHK). Metabolites from the natural actinorhodin producer *S. coelicolor* (abbreviated Strep) was used as a standard (positive control) for comparing metabolites produced in *wt* and engineered yeast strains: TC-3, TC-158, TC-175, TC-177. Red vertical lines on chromatograms depict the peaks of listed compounds based on known mass and positive control. Intensities of the peaks and elution times are shown on the corresponding axis.

The main goal of this study was to create a versatile and programmable platform for production of bacterial aromatic polyketides. To prove that our polyketide production platform can be engineered to express different polyketide synthesis modules, we replaced several key Act enzymes with enzymes from other *Streptomyces* species to achieve production of desired products (Fig. 4A). For this purpose, we reconstructed the Act pathway in yeast so that ActVI-2 (dehydrogenase), ActVI-4 (dehydrogenase), ActVA-5 (hydroxylase) and ActVB (flavin: NADH oxidoreductase) from the Act pathway were replaced with enzymes Med-9 (dehydrogenase), Med-29 (dehydrogenase), Med-7 (oxygenase) and Med-13 (oxidoreductase), respectively, from the medermycin biosynthesis pathway (Med pathway; NCBI GenBank: AB103463.1) [[36], [37], [38]] (Fig. 4A). Next, we first phenotypically assessed the reprogrammed yeast strains (Supplementary Fig. 1; TC-175, TC-177), which provided an indication if the enzymes can be functionally replaced in the platform strains. Further, we investigated production of desired compounds with comparative metabolite profiling by LC-MS. LC-MS analysis revealed that detected metabolites are indeed the Med pathway intermediates in both strains with 2–4 enzymatic steps replaced by enzymes from Med pathway (Fig. 4B; TC-175, TC-177). These results indicate that the developed platform system can be potentially reprogrammed and employed for production of diverse bacterial polyketide compounds.

### 3.6 Structural elucidation and confirmation of Act metabolites in yeast

Since none of the analytical standards for Act metabolites are commercially available and comparative metabolomics by LC-MS (using *S. coelicolor* metabolite extracts as standards) can only putatively identify Act metabolites produced in yeast, we aimed to elucidate the structure of produced compounds by nuclear magnetic resonance (NMR). After scale-up, we searched the chromatogram of the crude extract for peaks with a UV pattern matching that of actinorhodin. We focused on the most dominant peak with this UV, which we collected and subjected to an additional two rounds of HPLC purification. We isolated a peak with elution time of 3.2 min after the third round of HPLC and determined its exact mass to be *m/z* 297.0404 ([M − H]^-^), which corresponds to the molecular formula C_16_H_10_O_6_. This is the same molecular formula as the intermediate DMAC (Supplementary Fig. 8) [32]. This peak is present only in the chromatogram from the engineered yeast with expressed Act pathway; it is not present in the chromatogram of the wild-type yeast control extracted under identical conditions (Supplementary Fig. 9). We subsequently obtained NMR and IR data and confirmed that the molecule is DMAC (Supplementary Figs. 10–22 and Supplementary Table 5). It has been previously shown that DMAC is a shunt product from the Act gene cluster and it usually forms when ActVI-1 (ketoreductase) is not expressed [32]. Since DMAC is a major compound that is produced in yeast from the Act biosynthetic pathway, this indicates that not all pathway enzymes are optimally functioning, namely ActVI-1. However, because we have tentatively detected metabolites that are further in the pathway such as DHK, we believe that ActVI-1 is functional, but not optimally. Such an accumulation of a shunt product probably also results in low productivity of the later pathway metabolites such as DHK or even no production of actinorhodin.

## 4 Discussion and conclusions

In summary, we have developed a functional first-of-its-kind eukaryotic production platform for bacterial polyketide derived products by employing a plant type III polyketide synthase to produce compounds originally found only in bacteria. As a proof-of-concept we engineered and optimized our platform in *S. cerevisiae* to successfully produce a compound identified as bioactive bacterial polyketide DHK, but also a major shunt product DMAC which should be mitigated to improve production of downstream Act cluster products in future studies. Finally, we further demonstrated programmability of our system by replacing key enzymes in Act pathway with enzymes from different *Streptomyces* species to produce desired products. Such characterisation confirms systems ability to functionalize and produce octaketide derived products in a well-described, eukaryotic production workhorse. We further envision our platform to be useful for production of many novel compounds, including novel antibiotics, characterisation and functionalization of them, and moreover, sustainable production through cell factories.

## Declaration of competing interest

Jay D. Keasling has commercial interests in Amyris, Lygos, Demetrix, Napigen, Apertor Labs, Berkeley Brewing Sciences, and Ansa Biosciences.
