# Supplementary material for: Programmable polyketide biosynthesis platform for production of aromatic compounds in yeast
Source: Synth Syst Biotechnol. 2020 Jan 23;5(1):11–8. doi: 10.1016/j.synbio.2020.01.004 (PMC6992897; doi:10.1016/j.synbio.2020.01.004)
Supplement: Multimedia component 1 [file mmc1.docx]

**Programmable polyketide biosynthesis platform for production of aromatic compounds in yeast**

Tadas Jakočiūnas^1*^, Andreas K. Klitgaard^1^, Eftychia Eva Kontou^1^, Julie Bang Nielsen^1^, Emil Thomsen^1^, David Romero-Suarez^1^, Kai Blin^1^, Christopher J. Petzold^2^, Jennifer W. Gin^2^, Yaojun Tong^1^, Charlotte Held Gotfredsen^6^, Pep Charusanti^1^, Rasmus J.N. Frandsen^7^, Tilmann Weber^1^, Sang Yup Lee^1,8^, Michael K. Jensen^1^, Jay D. Keasling^1-5*^

^1^ The Novo Nordisk Foundation Center for Biosustainability, Technical University of Denmark, Kgs. Lyngby, Denmark

^2^ Joint BioEnergy Institute, Emeryville, CA, USA

^3^ Biological Systems & Engineering Division, Lawrence Berkeley National Laboratory, Berkeley, CA, USA

^4^ Department of Chemical and Biomolecular Engineering & Department of Bioengineering University of California, Berkeley, CA, USA

^5^ Center for Synthetic Biochemistry, Institute for Synthetic Biology, Shenzhen Institutes for Advanced Technologies, Shenzhen, China

^6^ Department of Chemistry, Technical University of Denmark, Kgs. Lyngby, Denmark

^7^ Department of Biotechnology and Biomedicine, Technical University of Denmark, Kgs. Lyngby, Denmark

^8^ Department of Chemical and Biomolecular Engineering (BK21 Plus Program), Korea Advanced Institute of Science and Technology (KAIST), 291 Daehak-ro, Yuseong-gu, Daejeon, 34141, Republic of Korea

*Address correspondence to Tadas Jakočiūnas: [tajak@biosustain.dtu.dk](mailto:tajak@biosustain.dtu.dk); Jay D. Keasling: [jdkeasling@lbl.gov](mailto:jdkeasling@lbl.gov)


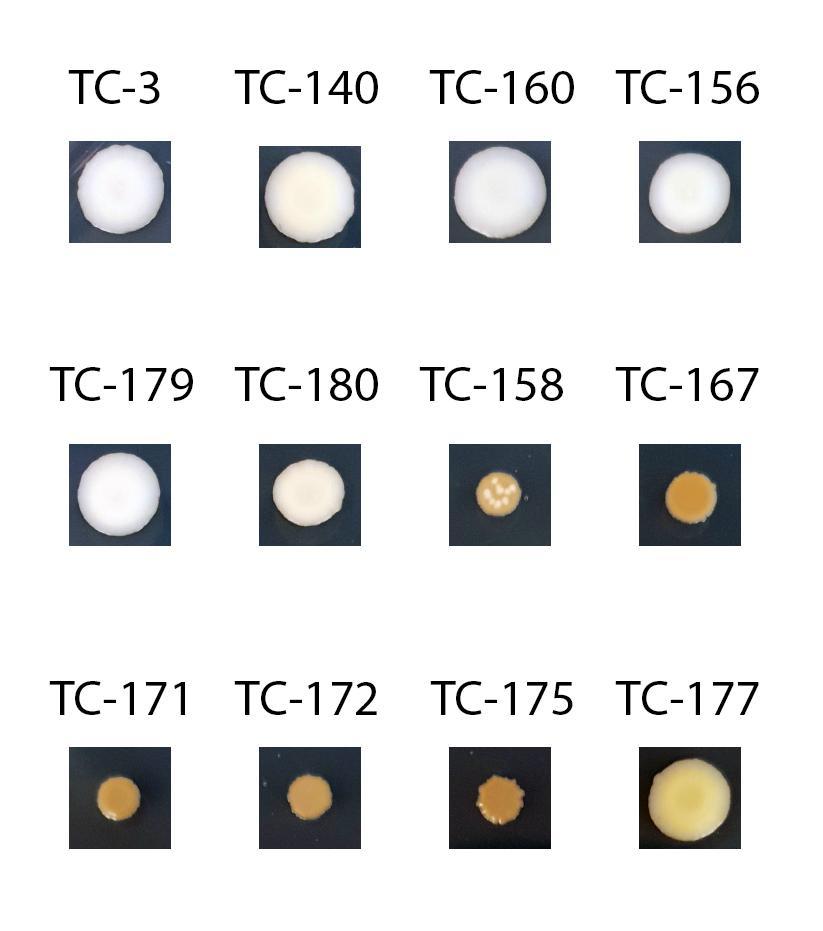


Supplementary Fig. 1. **Phenotypic comparison of constructed platform strains.**


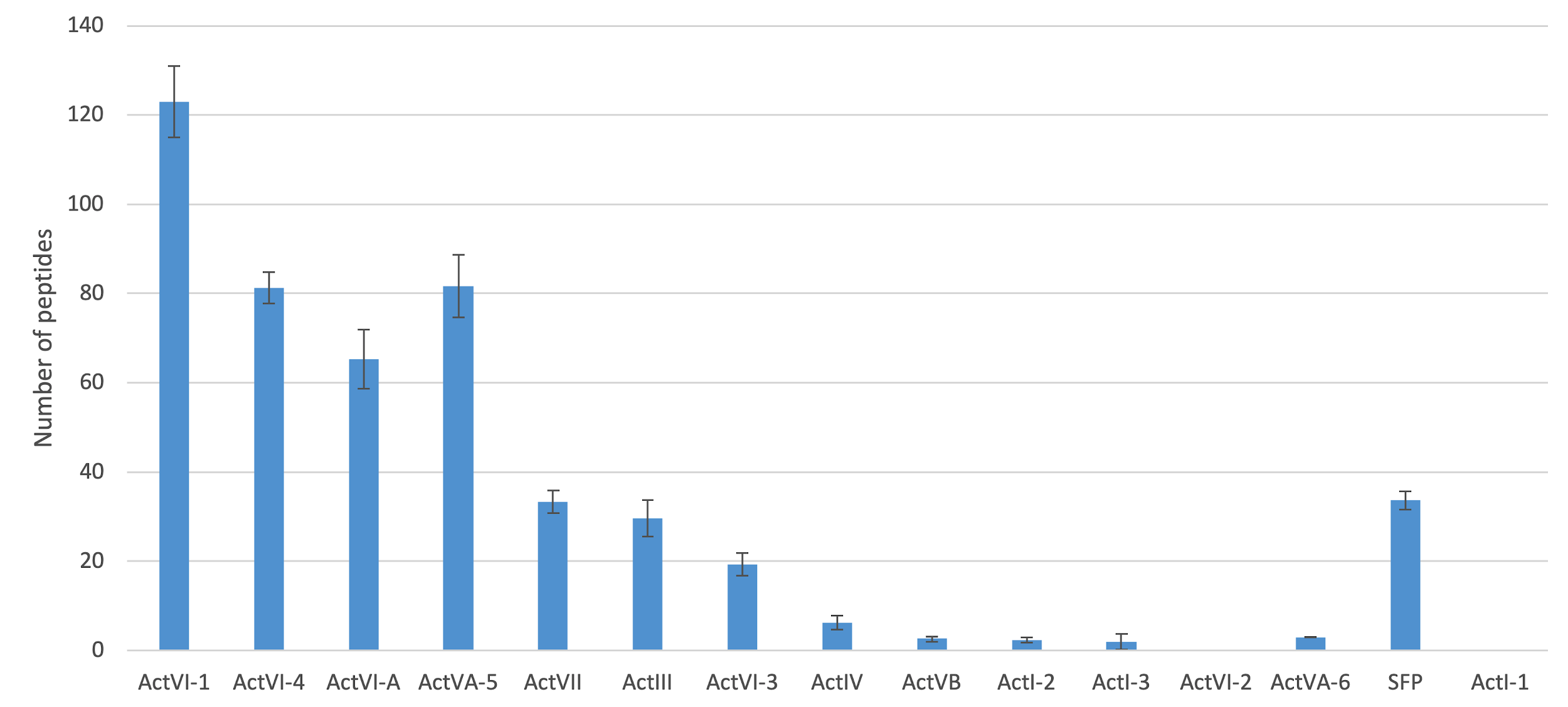
Supplementary Fig. 2. **Whole cell proteomics.**

Whole cell proteomics from strain TC-140 (expressing Act miniPKS and Act pathway). The data is based on 3 independent samples.

**A B**
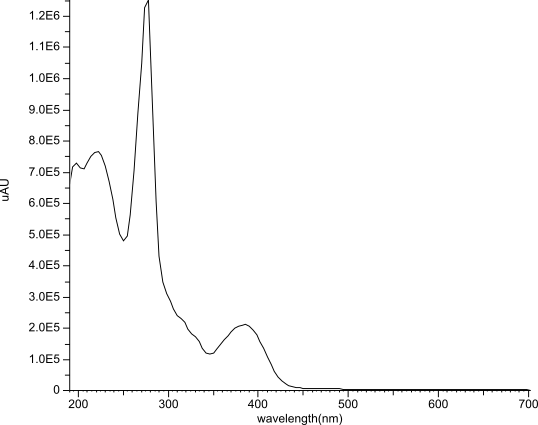


**
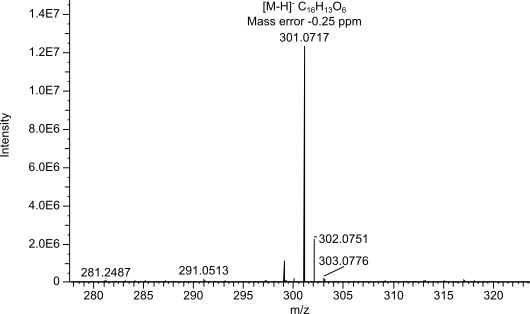
**

**C**


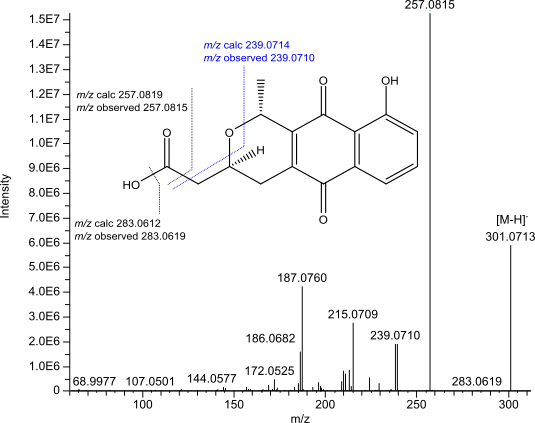


Supplementary Fig. 3. **Analysis of DHK by UV and MS/MS analyses.**

(A) Mass spectrum from the compound tentatively identified as the antibiotic - dihydrokalafungin (DHK). (B) UV spectrum from the compound tentatively identified as the antibiotic - dihydrokalafungin (DHK). (C) MS/MS spectrum from the compound tentatively identified as the antibiotic - dihydrokalafungin (DHK) with major fragments annotated. The MS/MS spectrum of DHK from yeast engineered strains matched with DHK MS/MS spectrum from *S. coelicolor.*


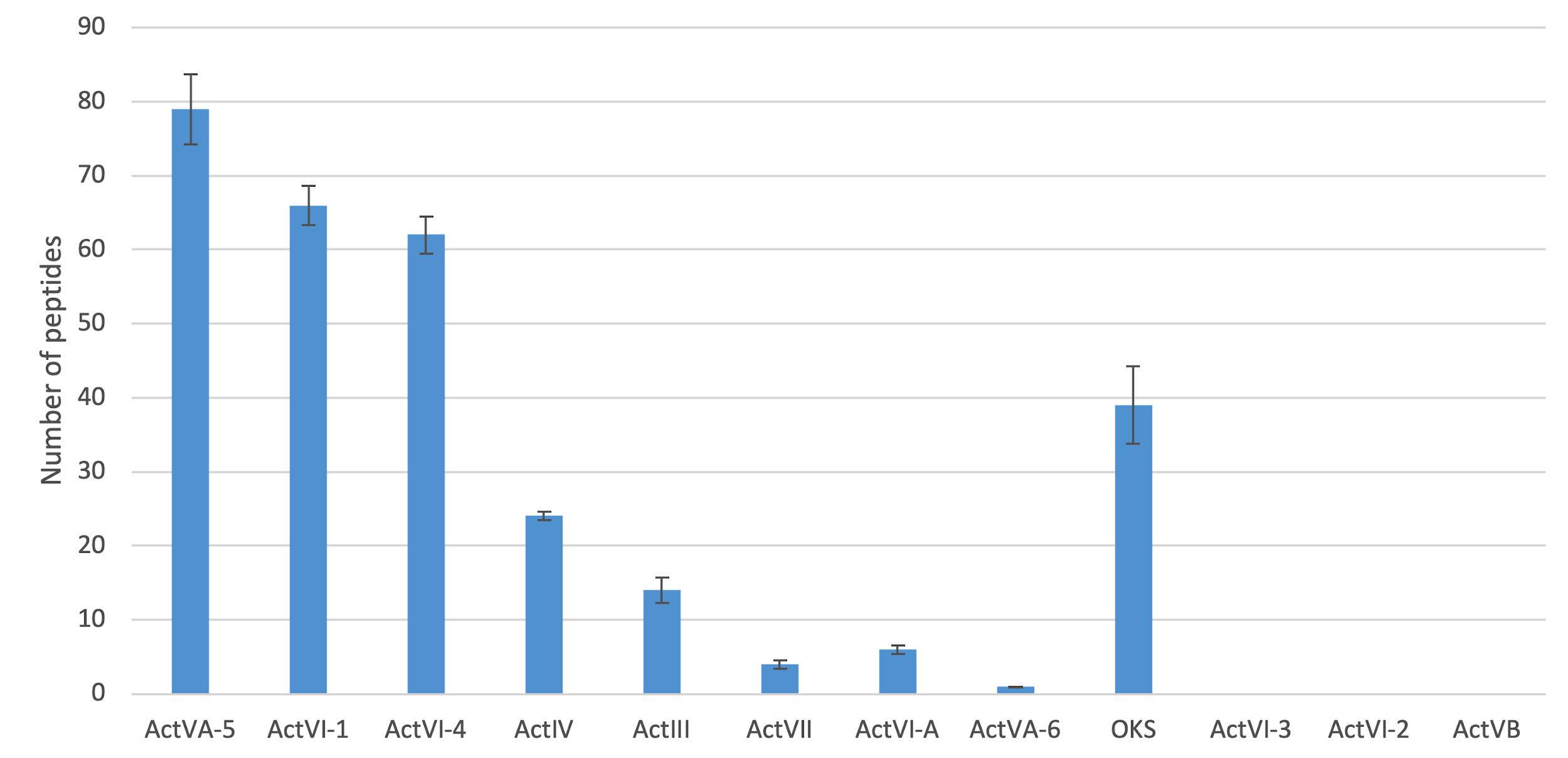


Supplementary Fig. 4. **Whole cell proteomics.**

Whole cell proteomics from strain TC-158 (expressing AaOKS and Act pathway). The data is based on 3 independent samples.


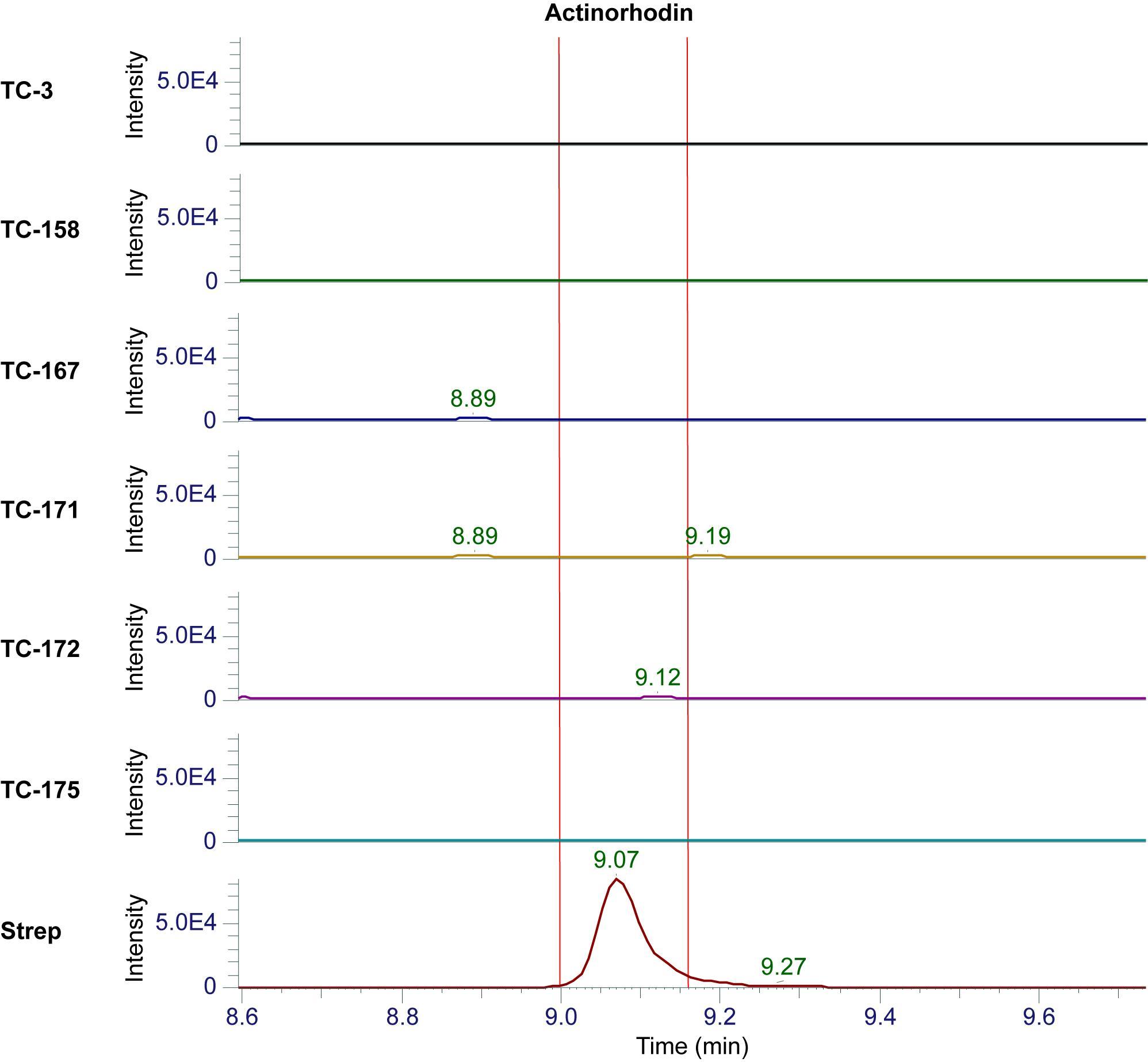


Supplementary Fig. 5. **Comparative metabolomics by LC-MS to evaluate the production of actinorhodin in engineered yeast strains.**


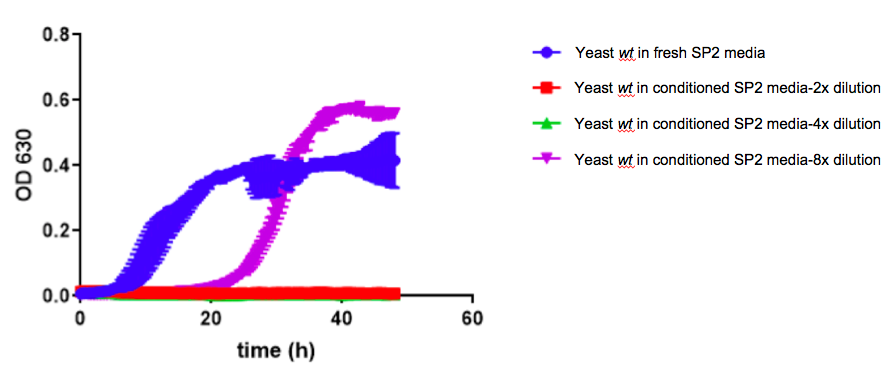


Supplementary Fig. 6. **Growth profiling of yeast *wt* strain in fresh and conditioned ISP2 media.** Conditioned media was prepared by growing *Streptomyces coelicolor* in ISP2 media for 168 hours, following removal of cells by centrifugation and addition of 2% glucose. Data shown from 3 independent replicates**
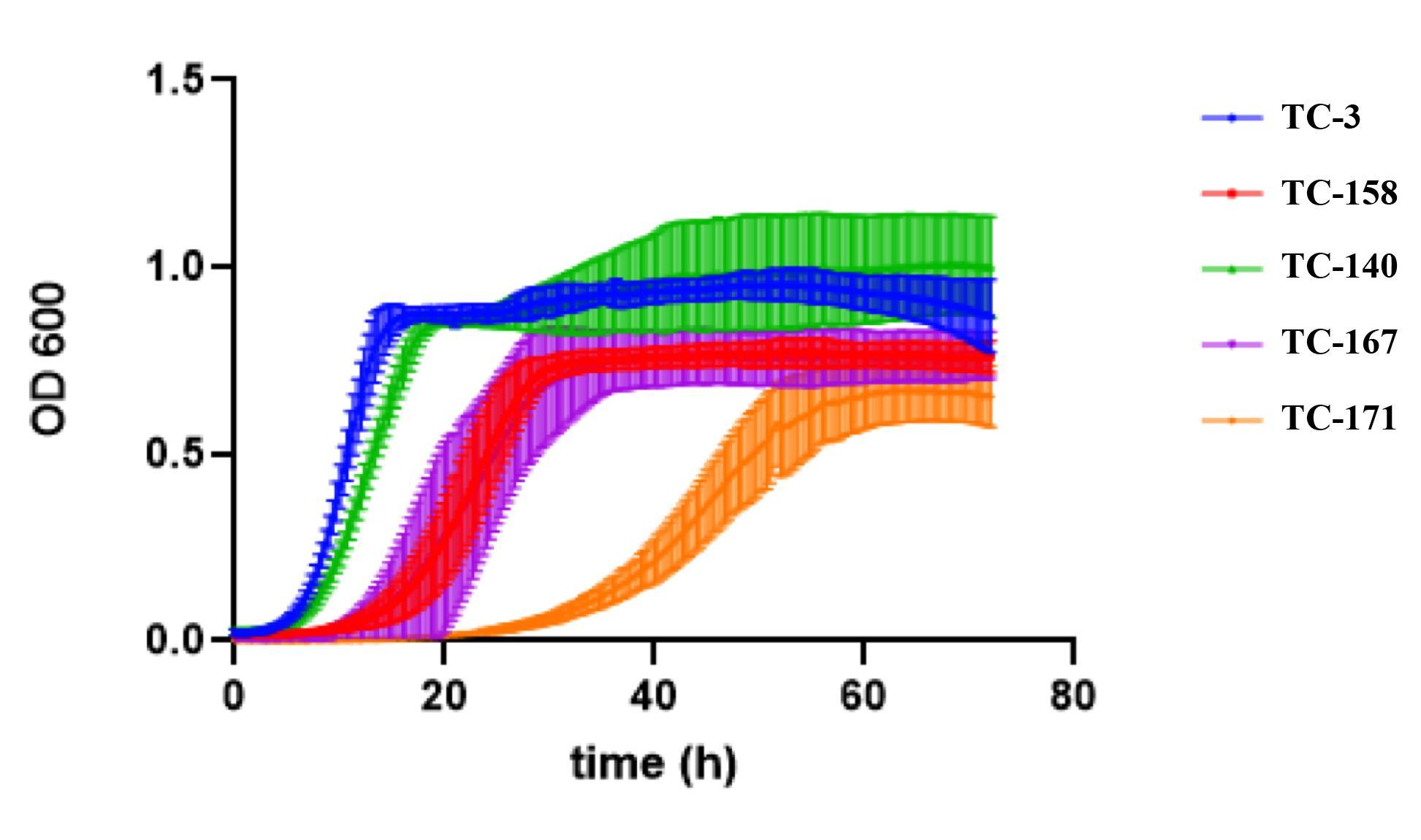
**

Supplementary Fig. 7. **Growth profiling of engineered yeast strains.**

TC-3 is *wt* control; TC-140 is strain with Act pathway expressing Act miniPKS; TC-158 strain expressing Act pathway and AaOKS; TC-167 is strain expressing AaOKS and optimized Act pathway; TC-171 is stain expressing AaOKS with Act pathway, including dimerize ActVA-4 (potential actinorhodin producer). Data shown from 3 independent replicates. Cells were grown in Synthetic Complete (SC) medium.


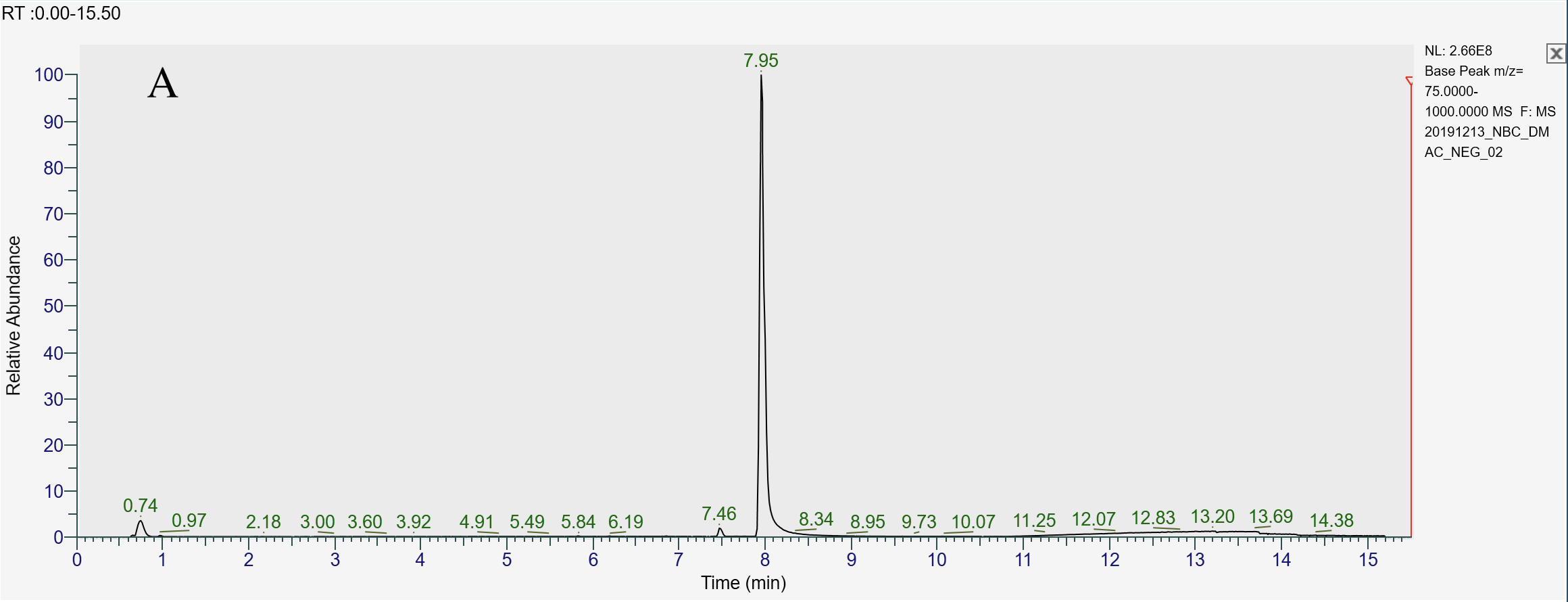


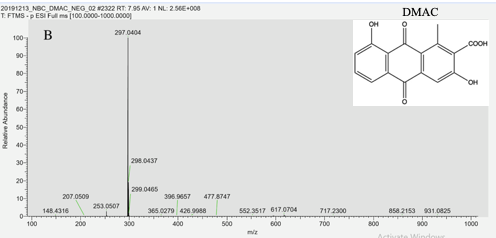


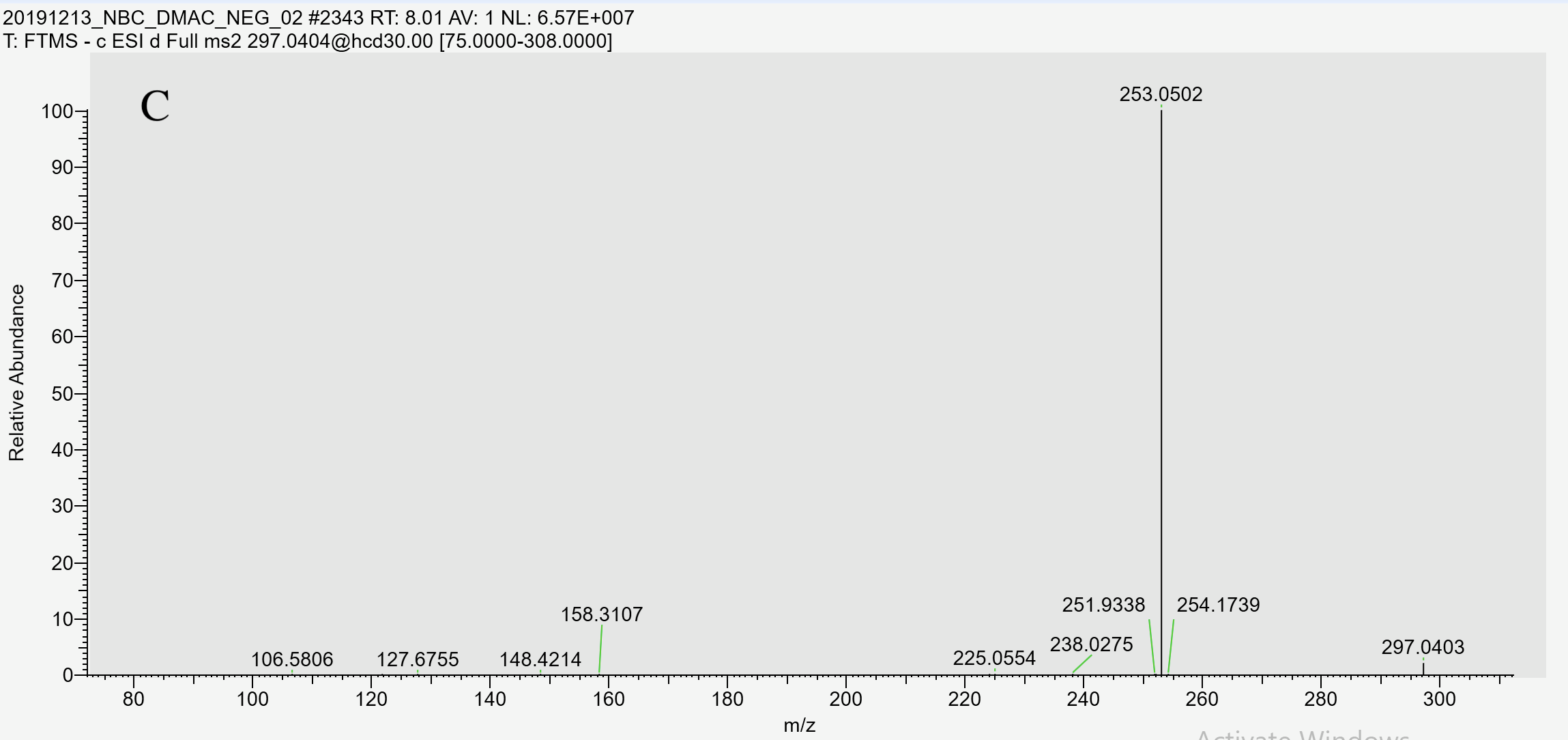


Supplementary Fig. 8. **MS and MS/MS analyses of the putative DMAC peak.**

DMAC was isolated by subjecting the crude sample to three rounds of HPLC purification. The DMAC peak eluted at 3.2 min after the third round. This peak was collected and injected into a separate UHPLC system coupled to an Orbitrap MS to assess purity and to obtain the high resolution mass (A) Chromatogram after injection into the UHPLC. There is a single dominant peak at 7.95 min. (B) Mass spectrum in negative ionization mode at 7.95 min. The most abundant ion has *m/z* of 297.0404 (∆ ppm 3.50) and a suggested molecular formula of C_16_H_10_O_6_, which is consistent with DMAC. (C) Tandem mass spectrometry in negative ionization mode of the molecular ion with *m/z* 297.0404. Fragmentation of the ion with *m/z* 297.0404 leads to the formation of an ion with *m/z* 253.0502 which is in agreement with the loss of the carboxylic acid in DMAC. This signal is also present in the mass spectrum, indicating that DMAC readily undergoes this fragmentation.


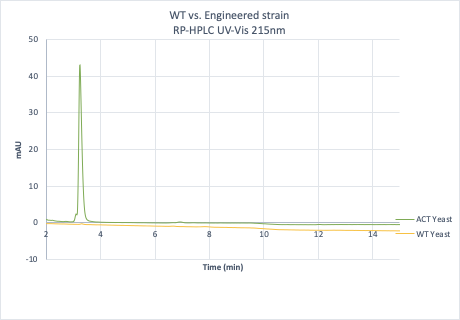

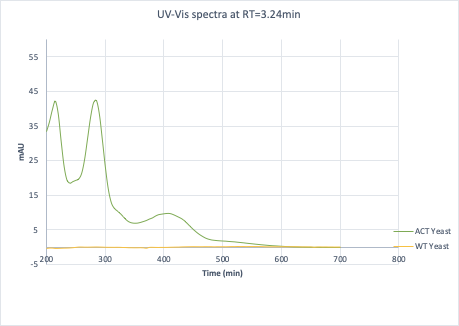


Supplementary Fig. 9. **HPLC chromatograms of extracts from wild-type yeast (yellow) and the yeast expressing the ACT cluster (green).**

The peak at 3.32 min is only present in the extract from the engineered strain and has absorbance bands consistent with that of anthraquinones [[1]](https://paperpile.com/c/7x3jzt/NtSW). The extract of the wild-type strain does not contain any UV active compounds in this retention time.

**Supplementary results**

**NMR data acquisition and analyses**

1H-NMR yielded five proton signals, one methyl-group, a singlet with integral around three, and four aromatic protons, two doublets, one triplet and one singlet all with an integral of around one.

The HSQC experiment revealed the correlation carbon to proton, for the methyl group deltaH = 3.85 ppm, deltaC = 20.94 ppm, and from the HMBC, the surrounding carbons from the benzene ring was identified as deltaC = 119.2 ppm, deltaC = 150.47 ppm (being the carbon the methyl group is attached to) and deltaC = 124.48 ppm. The aromatic proton identified as a singlet had the correlation from carbon to proton found from the HSQC, shifts deltaH = 7.23 ppm and deltaC = 115.95 ppm. The surrounding carbons identified from HMBC is as follows, deltaC = 183.5 ppm (C=O) and deltaC = 119.2 ppm (same peak as observed for the methyl-group). A DQF-COSY experiment and H2BC experiment, reveals that the remaining aromatic protons, arrive from the same spin system. The first doublet has the shifts deltaH = 7.60 ppm and deltaC = 135.69, from the DQF-COSY spectra a correlation to the proton deltaH = 7.67 ppm and from H2BC the nearest neighboring carbon shift deltaC = 118.26 ppm, corresponding to the aromatic proton with triplet splitting. The surrounding carbons were deltaC = 118.26, deltaC = 124.72 ppm (the carbon that correlates to the last aromatic proton according to HSQC experiments), and deltaC = 183.5 ppm (C=O, same carbon correlating to the single aromatic proton). The aromatic proton forming a triplet had the shifts deltaH = 7.67 ppm and deltaC = 118.26 ppm, it correlates to both doublets in the DQF-COSY spectra and both of the carbons correlated to those protons. From the HMBC the surrounding carbons were deltaC = 161.9 ppm (phenol carbon C-OH), deltaC = 133.0 ppm. The last aromatic proton is a doublet and in DQF-COSY and H2BC correlates triplet proton and the correlated carbon. The HMBC shows correlation to the surrounding carbons, deltaC = 161.9 ppm (phenol carbon, C-OH) and deltaC = 118.26 ppm.


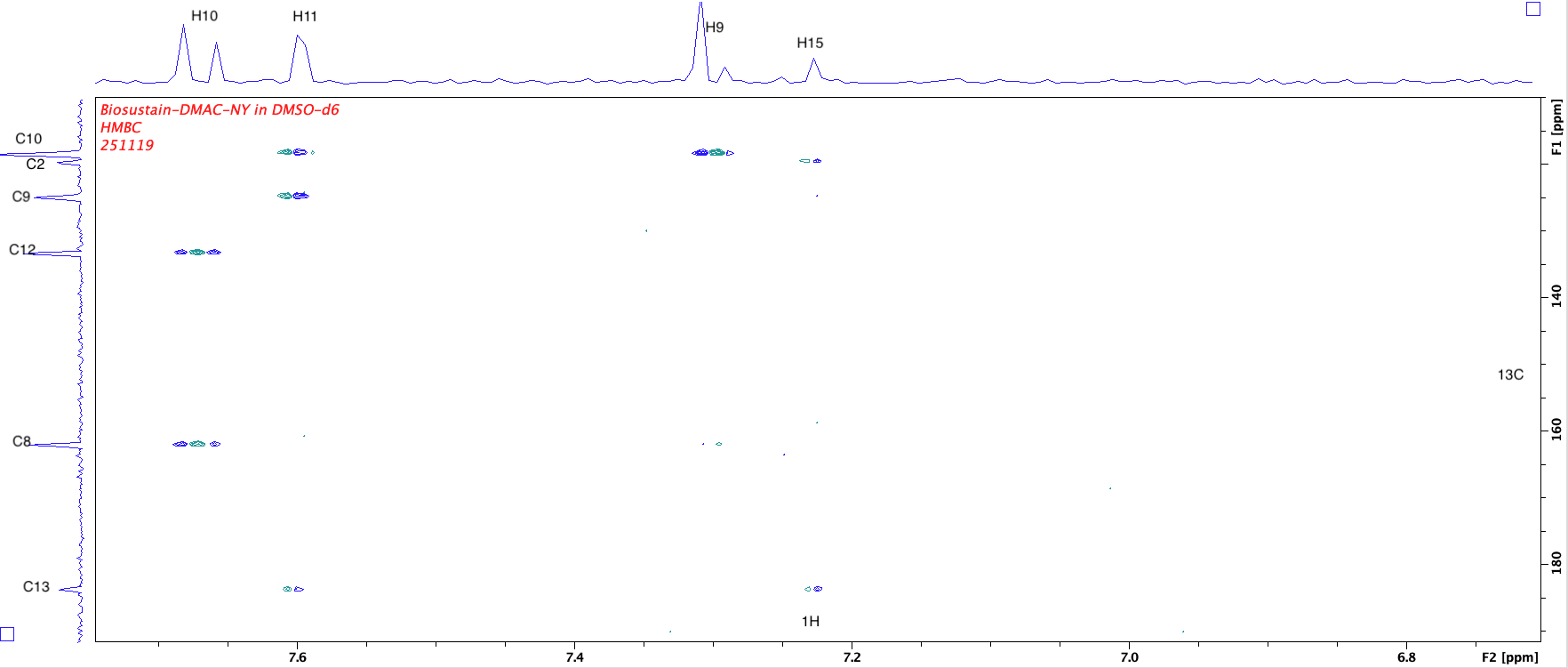


Supplementary Fig. 10. HMBC spectra zoomed-in for protons with shifts in the range for 6.7 ppm to 7.75 ppm and carbon with shifts in the range 110 ppm to 190 ppm. The spectra shows the correlations for the protons directly bound to the benzene ring.


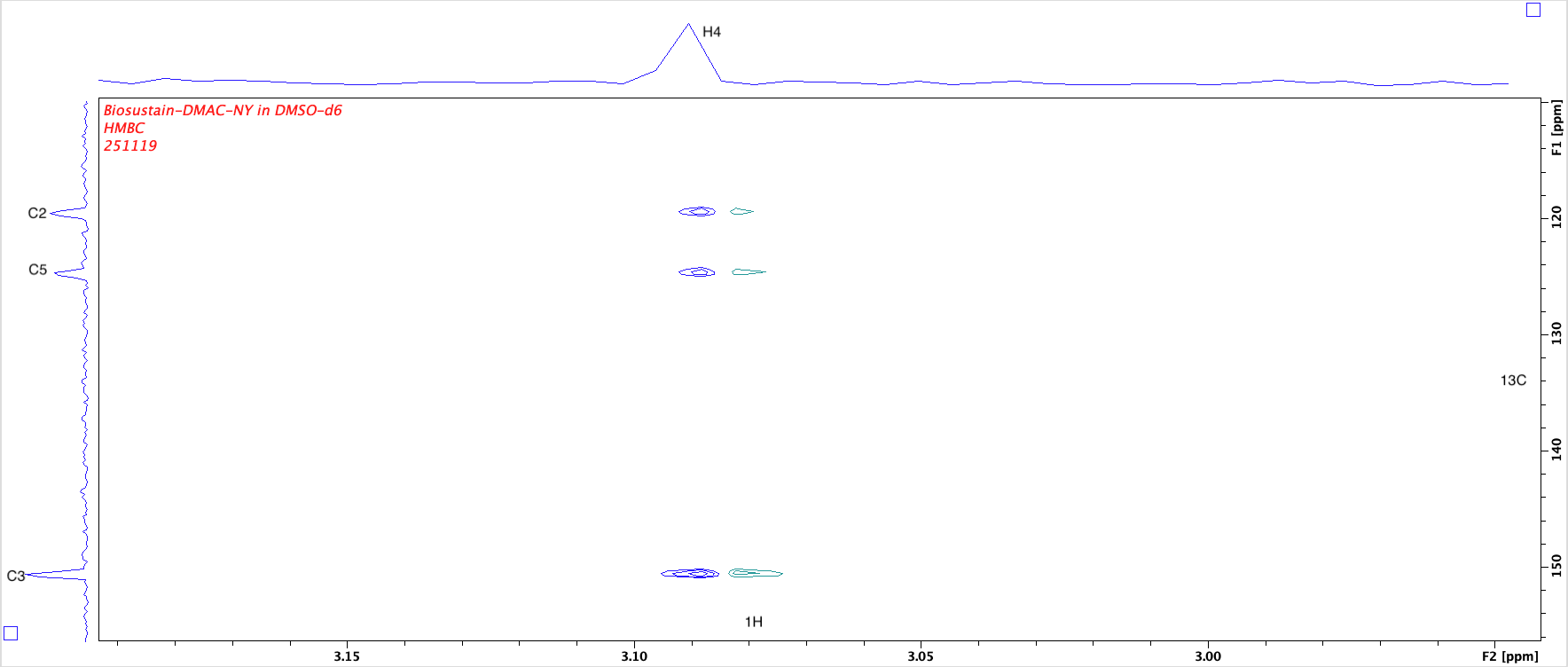


Supplementary Fig. 11. HMBC spectra zoomed-in for the protons at 3.85 ppm corresponding to methyl-group attached to a benzene ring, and the correlations to the surrounding it in the benzene ring.


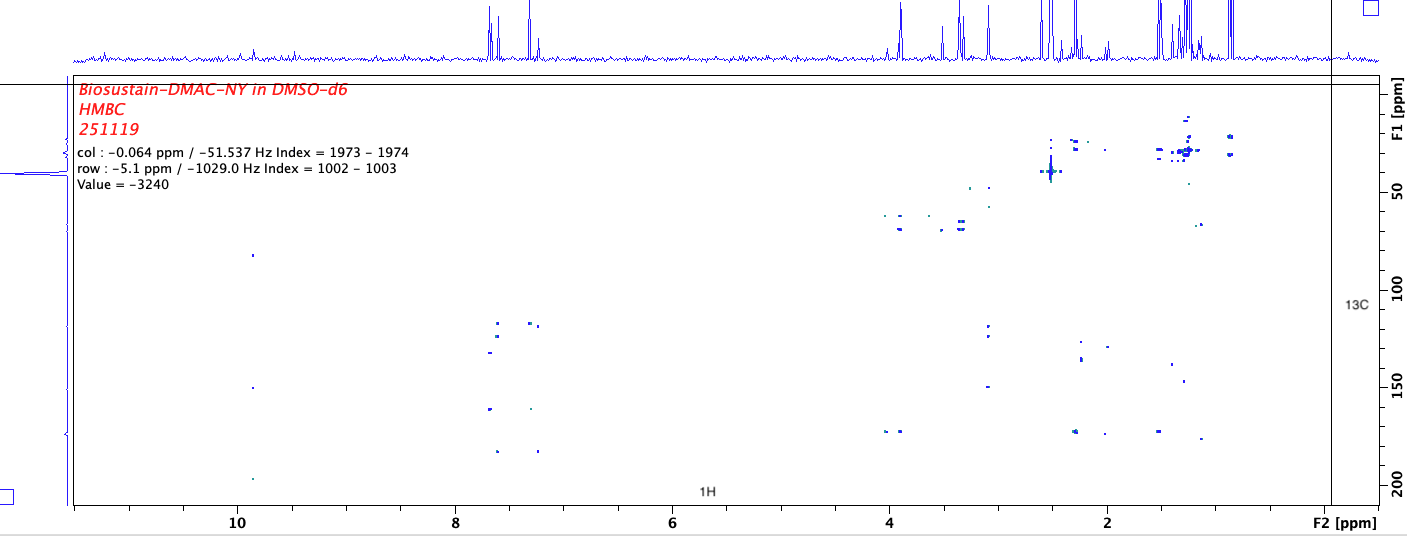


Supplementary Fig. 12. HMBC spectra

(A)


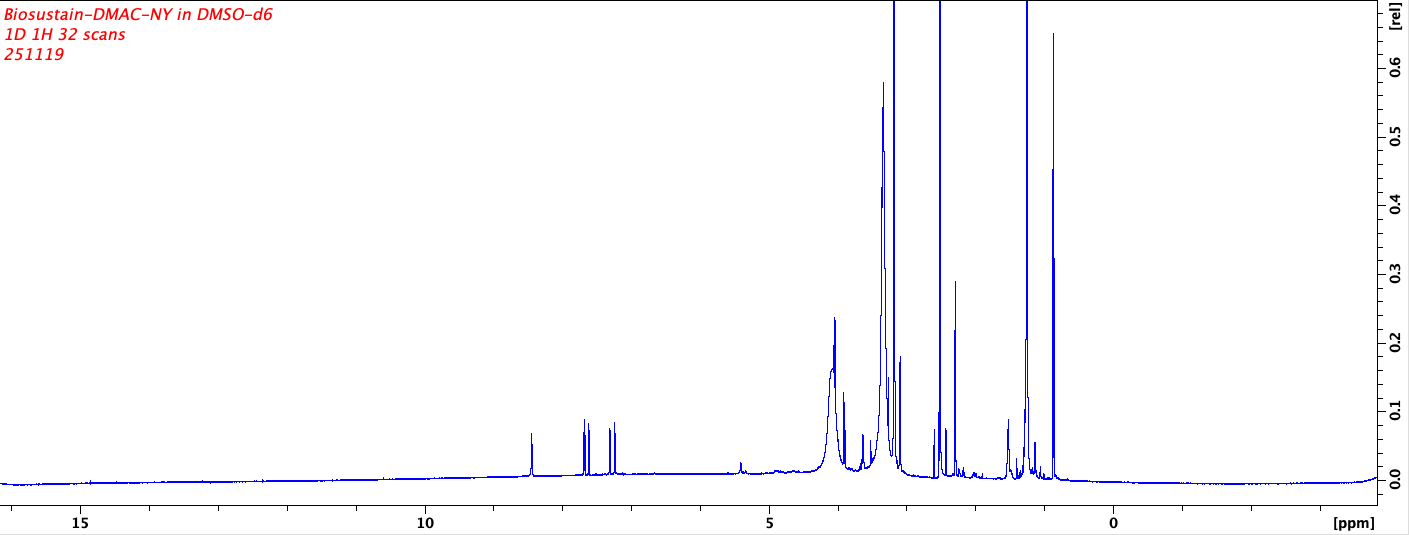


(B)


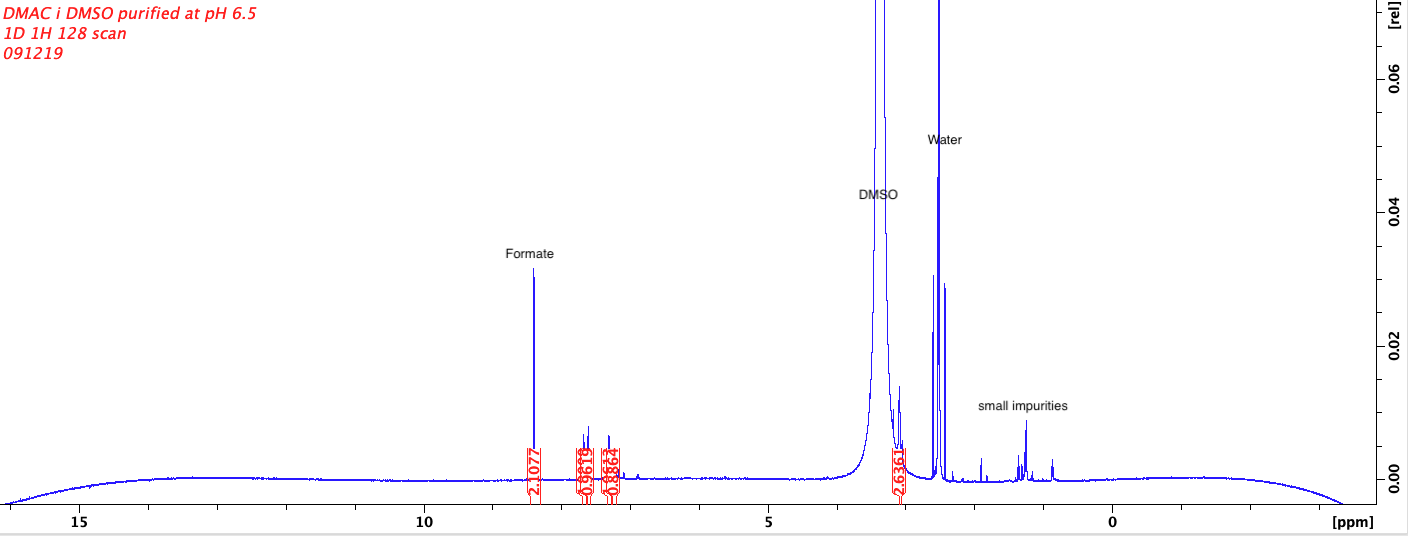


Supplementary Fig. 13. 1H-NMR obtained in DMSO-d6, in the lower spectra is seen a few impurities (A), but after another clean-up, separation on Xbridge-C18 column another 1H-NMR was obtained and the impurities are almost removed (B).


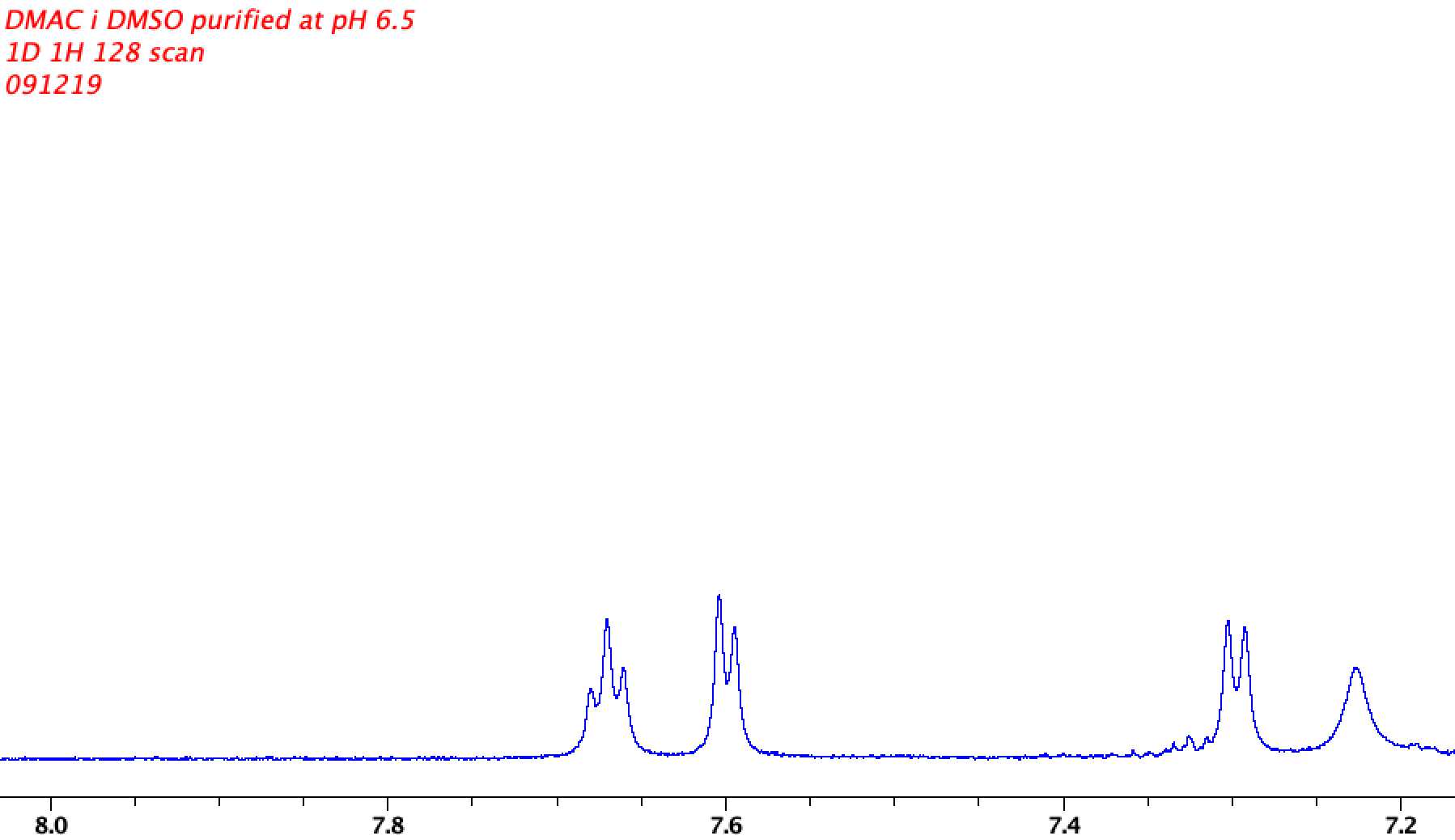


Supplementary Fig. 14. Zoomed-in on the peaks from the aromatic protons H9, H10, H11 and H15


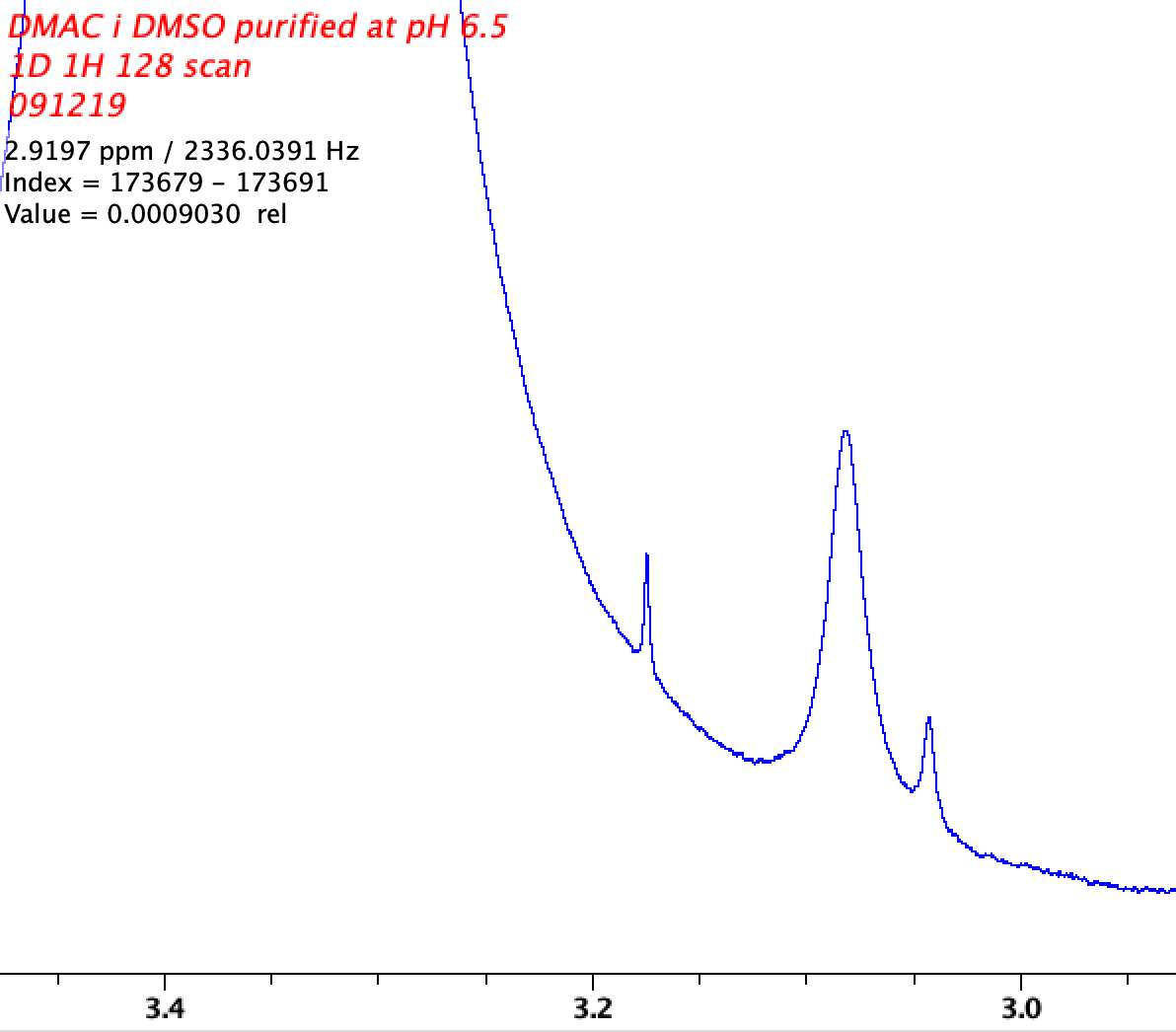


Supplementary Fig. 15. Zoomed-in on the peak from the methyl, close to the DMSO peak.


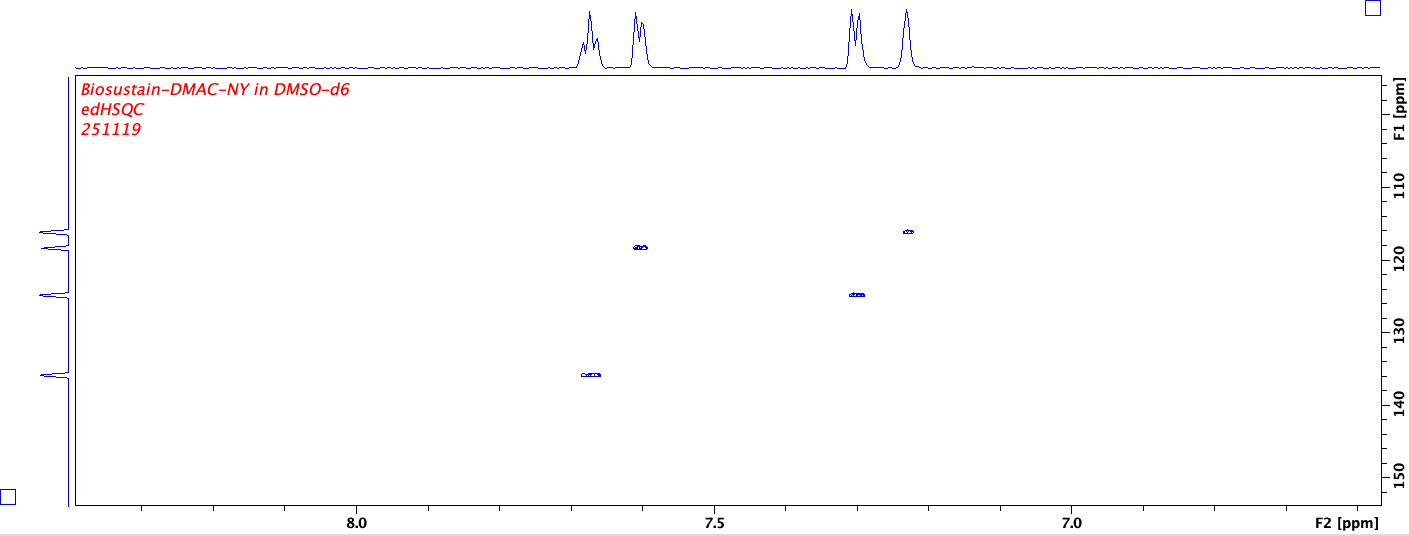


Supplementary Fig. 16. Ed-HSQC zoomed-in on the aromatic protons and the correlated carbons.


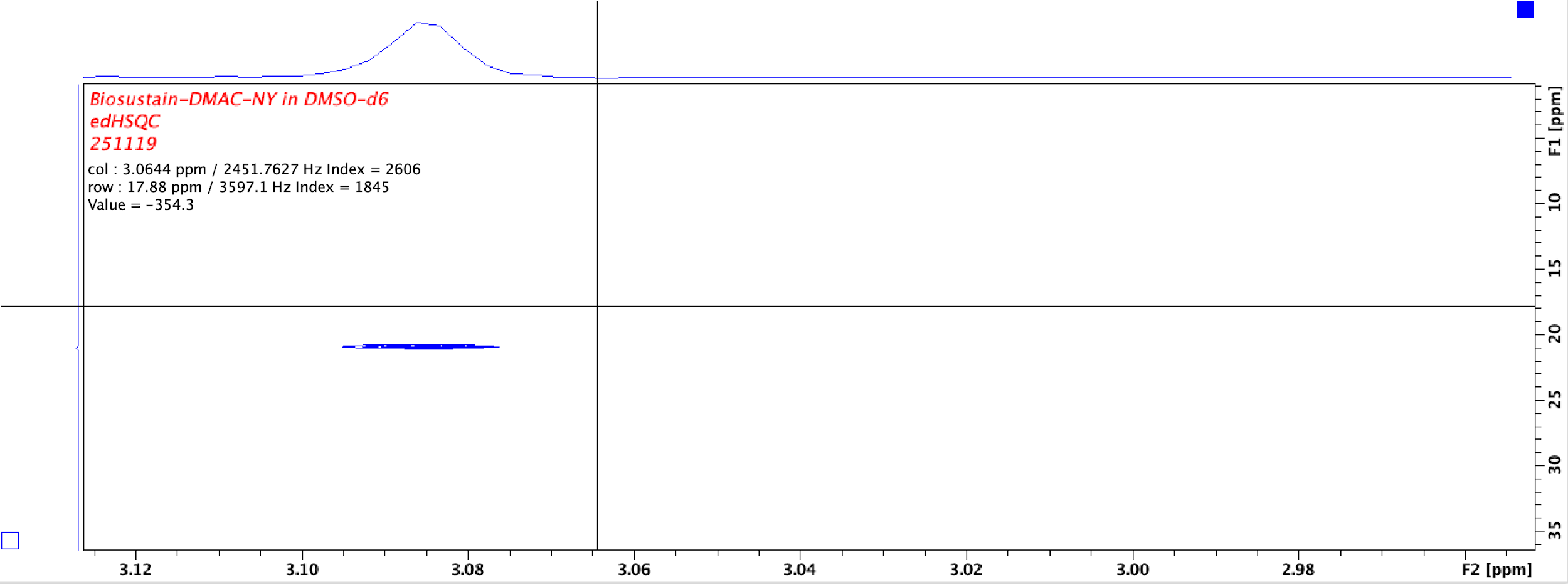


Supplementary Fig. 17. Ed-HSQC zoomed-in on the protons from the methyl group and the correlated carbon.


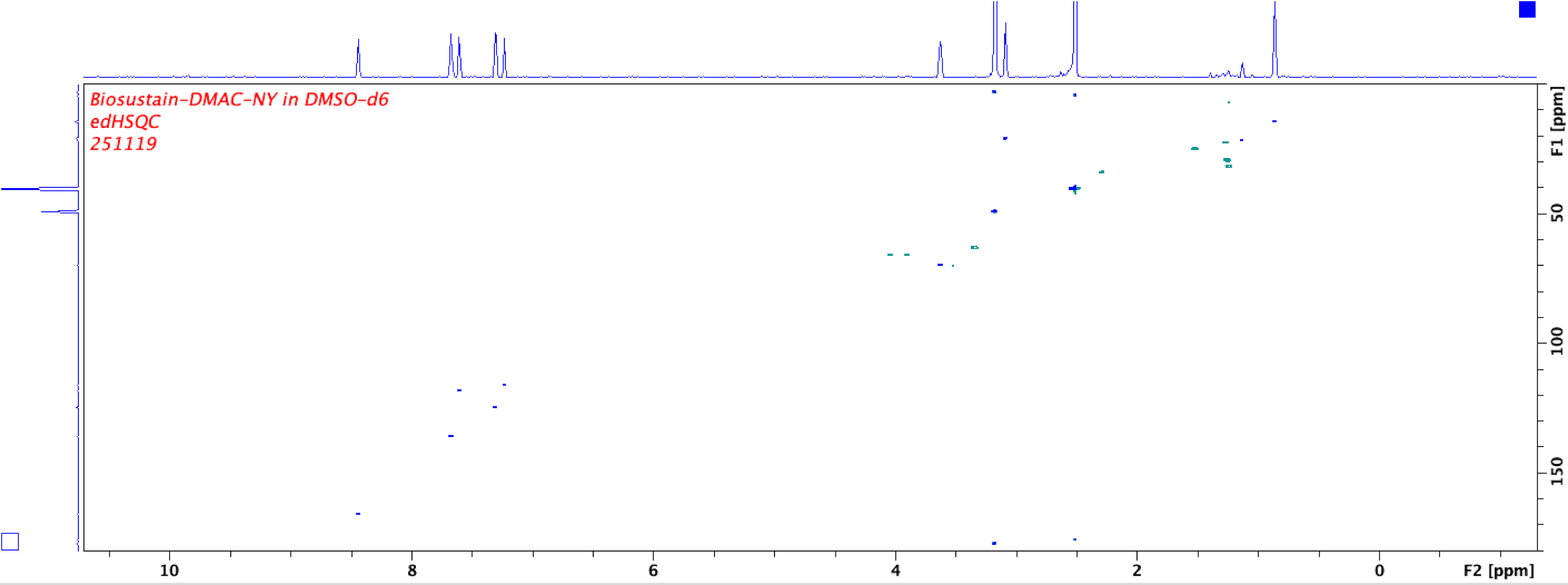


Supplementary Fig. 18. Ed-HSQC


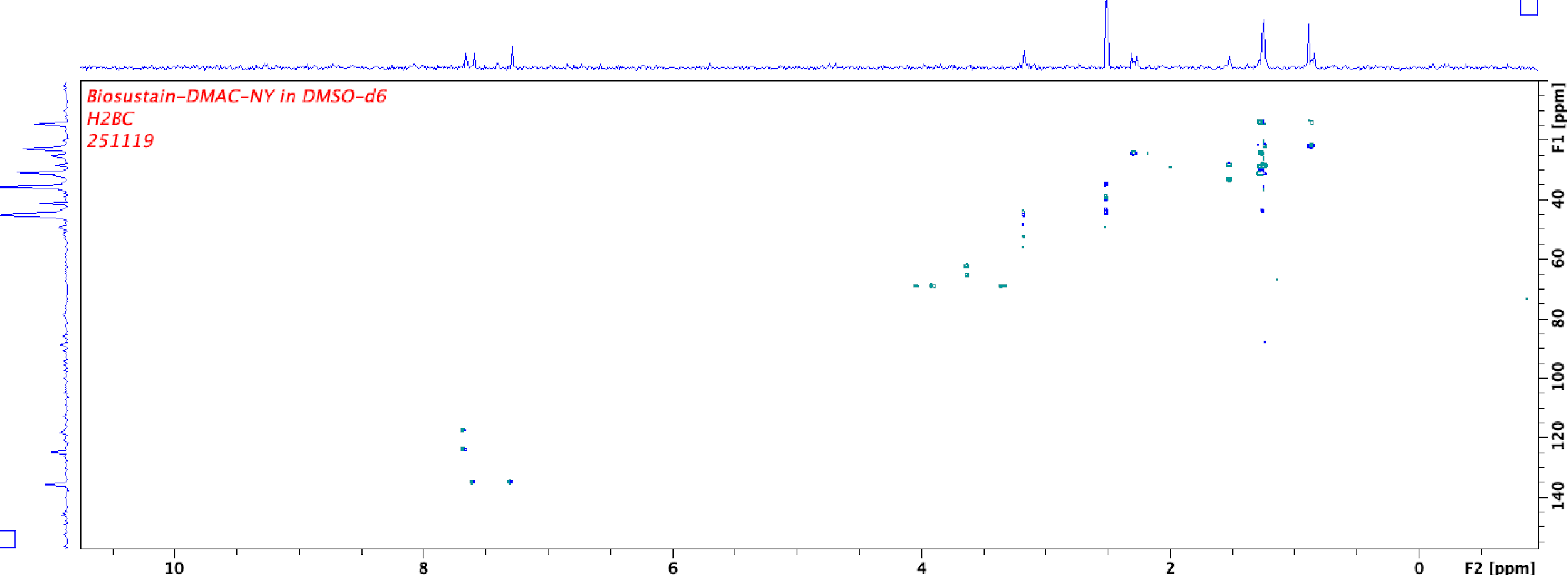


Supplementary Fig. 19. H2BC


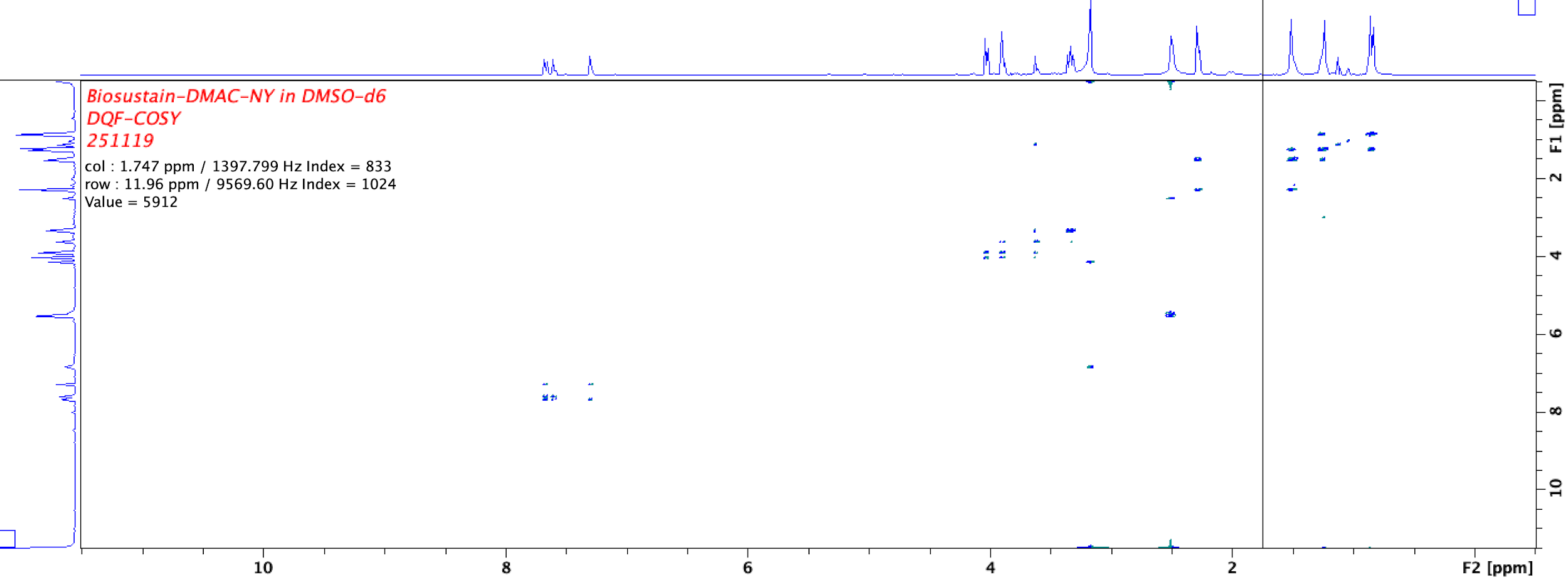


Supplementary Fig. 20. DQF-COSY


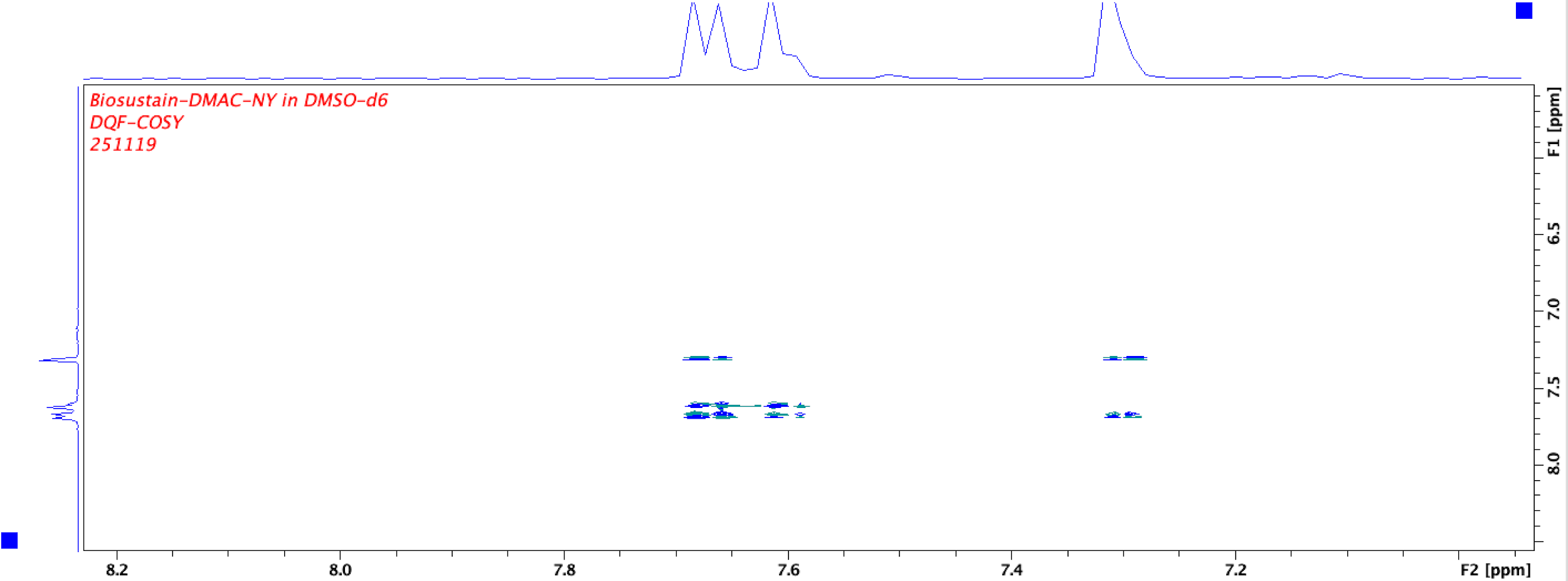


Supplementary Fig. 21. zoomed-in on DQF-COSY, in the aromatic protons region.


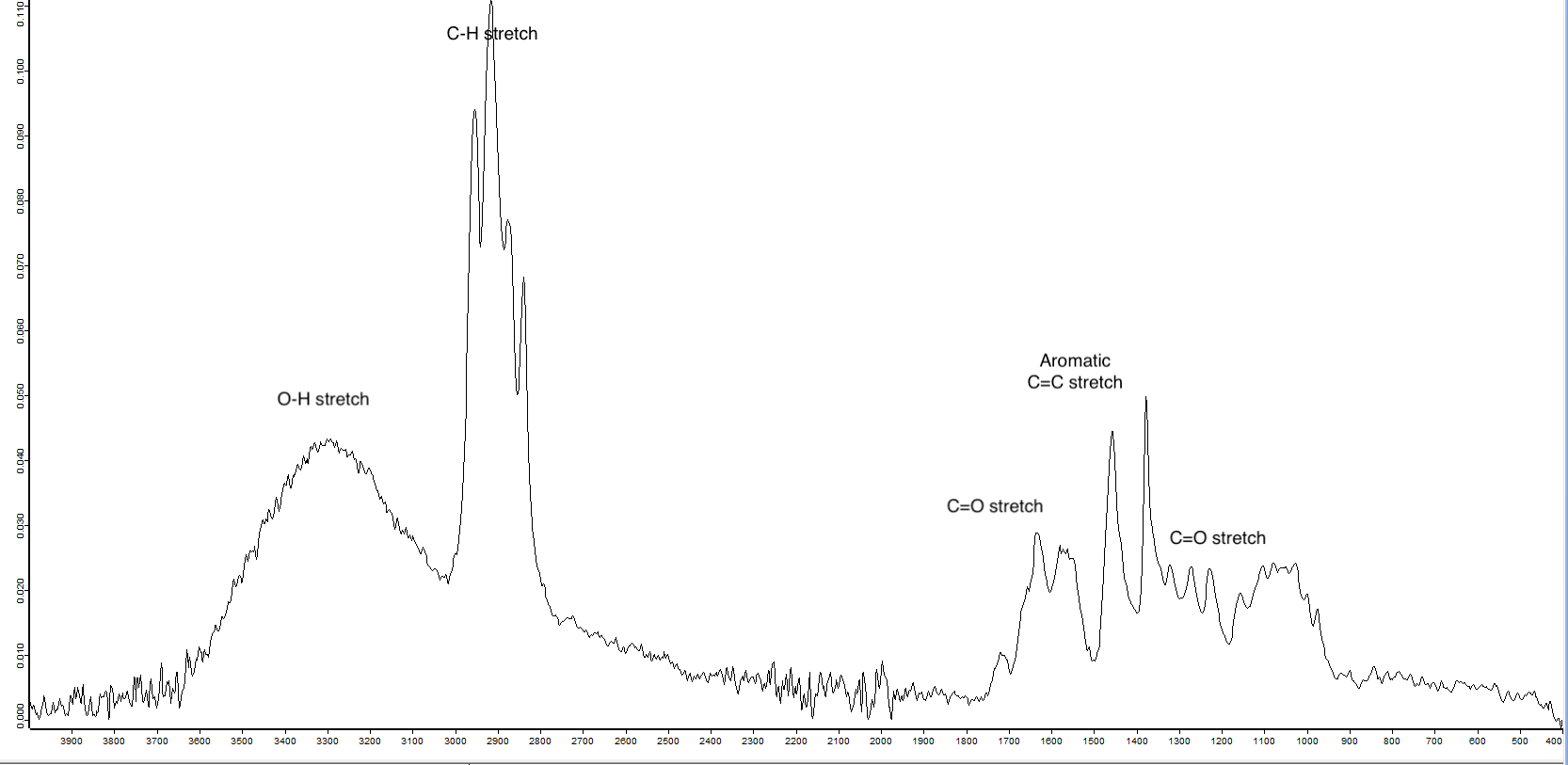


Supplementary Fig. 22. IR-spectroscopy

Phenol and carboxylic acid O-H stretch from 3650-3000, sp3 C-H stretch and aromatic sp2 C-H stretch in the range from 3000-2750. C=O stretch from carboxylic acid and from the ketone, moved from 1710-1715 to around 1650 cm^-1 due to the highly conjugated system surrounding it. Aromatic C=C stretch at 1600 cm^-1 and around 1450-1475 cm-1 were observed in the IR spectrum.

Supplementary Table 1. **Construction steps, expression units and genomic integration sites for platform strains TC-140 and TC-158 respectively.**

| **Step** | **Expression unit** | **Integration site** |
| --- | --- | --- |
| **Step 1** | tADH1-ActI-1 <-pTDH3-pTEF1-> ActI-3-tCYC1 | **XI-1** |
|  | tADH1-ActI-2 <-pTDH3-pTEF1-> SFP-tCYC1 | **XII-1** |
| **Step 2** | tADH1-ActIII <-pTDH3-pTEF1-> ActIV-tCYC1 | **X-3** |
|  | tADH1-ActVI-1 <-pTDH3-pTEF1-> ActVI-3-tCYC1 | **XI-2** |
|  | tADH1-ActVII <-pTDH3-pTEF1-> ActVI-A-tCYC1 | **XII-2** |
| **Step 3** | tADH1-ActVI-4 <-pTDH3-pTEF1-> ActVA-6-tCYC1 | **X-2** |
|  | pTEF1-> ActVI-2-tCYC1 | **XI-5** |
|  | tADH1-ActVA-5 <-pTDH3-pTEF1-> ActVB-tCYC1 | **XII-4** |

| **Step** | **Expression unit** | **Integration site** |
| --- | --- | --- |
| **Step 1** | pTDH3-> AaOKS-tCYC1 | **Expression from plasmid** |
| **Step 2** | tADH1-ActIII <-pTDH3-pTEF1-> ActIV-tCYC1 | **X-3** |
|  | tADH1-ActVI-1 <-pTDH3-pTEF1-> ActVI-3-tCYC1 | **XI-2** |
|  | tADH1-ActVII <-pTDH3-pTEF1-> ActVI-A-tCYC1 | **XII-2** |
| **Step 3** | tADH1-ActVI-4 <-pTDH3-pTEF1-> ActVA-6-tCYC1 | **X-2** |
|  | pTEF1-> ActVI-2-tCYC1 | **XI-5** |
|  | tADH1-ActVA-5 <-pTDH3-pTEF1-> ActVB-tCYC1 | **XII-4** |

Supplementary Table 2. **Strain table.**

| Name | Expression units | Integration sites | Plasmids |
| --- | --- | --- | --- |
| TC-3 | CEN.PK2-1C + PTEF1-Cas9 |  | pRS414-TEF1p-Cas9-CYC1t |
| TC-140 | ActI-1 <-PTDH3-PTEF1-> ActI-3;  ActI-2 <-PTDH3-PTEF1-> SFP;  ActIII <-PTDH3-PTEF1-> ActIV;  ActVI-1 <-PTDH3-PTEF1-> ActVI-3;  ActVII <-PTDH3-PTEF1-> ActVI-A;  ActVI-4 <-PTDH3-PTEF1-> ActVA-6;  PTEF1-> ActVI-2;  ActVA-5 <-PTDH3-PTEF1-> ActVB. | XI-1;  XII-1;  X-3;  XI-2;  XII-2;  X-2;  XI-5;  XII-4. | pTAJAK-122;  pTAJAK-123;  pTAJAK-131;  pTAJAK-132;  pTAJAK-133;  pTAJAK-134;  pTAJAK-135;  pTAJAK-136. |
| TC-156 | ActIII <-PTDH3-PTEF1-> ActIV;  ActVI-1 <-PTDH3-PTEF1-> ActVI-3;  ActVII <-PTDH3-PTEF1-> ActVI-A;  ActVI-4 <-PTDH3-PTEF1-> ActVA-6;  PTEF1-> ActVI-2;  ActVA-5 <-PTDH3-PTEF1-> ActVB. | X-3;  XI-2;  XII-2;  X-2;  XI-5;  XII-4. | pTAJAK-131;  pTAJAK-132;  pTAJAK-133;  pTAJAK-134;  pTAJAK-135;  pTAJAK-136. |
| TC-158 | ActIII <-PTDH3-PTEF1-> ActIV;  ActVI-1 <-PTDH3-PTEF1-> ActVI-3;  ActVII <-PTDH3-PTEF1-> ActVI-A;  ActVI-4 <-PTDH3-PTEF1-> ActVA-6;  PTEF1-> ActVI-2;  ActVA-5 <-PTDH3-PTEF1-> ActVB;  PTDH3->AaOKS. | X-3;  XI-2;  XII-2;  X-2;  XI-5;  XII-4. | pTAJAK-131;  pTAJAK-132;  pTAJAK-133;  pTAJAK-134;  pTAJAK-135;  pTAJAK-136;  pTAJAK-205. |
| TC-160 | ActIII <-PTDH3-PTEF1-> ActIV;  ActVI-1 <-PTDH3-PTEF1-> ActVI-3;  ActVII <-PTDH3-PTEF1-> ActVI-A;  ActVI-4 <-PTDH3-PTEF1-> ActVA-6;  PTEF1-> ActVI-2;  ActVA-5 <-PTDH3-PTEF1-> ActVB;  PTDH3->AaOKS. | X-3;  XI-2;  XII-2;  X-2;  XI-5;  XII-4  XI-3 | pTAJAK-131;  pTAJAK-132;  pTAJAK-133;  pTAJAK-134;  pTAJAK-135;  pTAJAK-136;  pTAJAK-204. |
| TC-167 | ActIII <-PTDH3-PTEF1-> ActIV;  ActVI-1 <-PTDH3-PTEF1-> ActVI-3;  ActVII <-PTDH3-PTEF1-> ActVI-A;  ActVI-4 <-PTDH3-PTEF1-> ActVA-6;  PTEF1-> ActVI-2;  ActVA-5 <-PTDH3-PTEF1-> ActVB;  PTDH3->AaOKS;  ActVI-2 <-PTDH3-PTEF1-> ActVA-6;  ActVB <-PTDH3-PTEF1-> ActVI-3. | X-3;  XI-2;  XII-2;  X-2;  XI-5;  XII-4;  XI-1;  XII-1. | pTAJAK-131;  pTAJAK-132;  pTAJAK-133;  pTAJAK-134;  pTAJAK-135;  pTAJAK-136;  pTAJAK-205;  pTAJAK-220;  pTAJAK-221. |
| TC-171 | ActIII <-PTDH3-PTEF1-> ActIV;  ActVI-1 <-PTDH3-PTEF1-> ActVI-3;  ActVII <-PTDH3-PTEF1-> ActVI-A;  ActVI-4 <-PTDH3-PTEF1-> ActVA-6;  PTEF1-> ActVI-2;  ActVA-5 <-PTDH3-PTEF1-> ActVB;  PTDH3->AaOKS;  PTDH3->ActVA-4. | X-3;  XI-2;  XII-2;  X-2;  XI-5;  XII-4;  XII-5 | pTAJAK-131;  pTAJAK-132;  pTAJAK-133;  pTAJAK-134;  pTAJAK-135;  pTAJAK-136;  pTAJAK-205;  pTAJAK-229. |
| TC-172 | ActIII <-PTDH3-PTEF1-> ActIV;  ActVI-1 <-PTDH3-PTEF1-> ActVI-3;  ActVII <-PTDH3-PTEF1-> ActVI-A;  ActVI-4 <-PTDH3-PTEF1-> ActVA-6;  PTEF1-> ActVI-2;  ActVA-5 <-PTDH3-PTEF1-> ActVB;  PTDH3->AaOKS;  ActVI-2 <-PTDH3-PTEF1-> ActVA-6;  ActVB <-PTDH3-PTEF1-> ActVI-3;  PTDH3->ActVA-4. | X-3;  XI-2;  XII-2;  X-2;  XI-5;  XII-4;  XI-1;  XII-1;  XII-5 | pTAJAK-131;  pTAJAK-132;  pTAJAK-133;  pTAJAK-134;  pTAJAK-135;  pTAJAK-136;  pTAJAK-205;  pTAJAK-220;  pTAJAK-221;  pTAJAK-229. |
| TC-175 | ActIII <-PTDH3-PTEF1-> ActIV;  ActVI-1 <-PTDH3-PTEF1-> ActVI-3;  ActVII <-PTDH3-PTEF1-> ActVI-A;  PTDH3->AaOKS;  Med-29 <-PTDH3-PTEF1-> ActVA-6;  Med-9 <-PTDH3-PTEF1-> ActVA-4;  ActVA-5 <-PTDH3-PTEF1-> ActVB. | X-3;  XI-2;  XII-2;  X-2;  XI-5;  XII-4. | pTAJAK-131;  pTAJAK-132;  pTAJAK-133;  pTAJAK-205;  pTAJAK-227;  pTAJAK-228;  pTAJAK-136. |
| TC-177 | ActIII <-PTDH3-PTEF1-> ActIV;  ActVI-1 <-PTDH3-PTEF1-> ActVI-3;  ActVII <-PTDH3-PTEF1-> ActVI-A;  PTDH3->AaOKS;  Med-29 <-PTDH3-PTEF1-> ActVA-6  Med-9 <-PTDH3-PTEF1-> ActVA-4  Med7 <-PTDH3-PTEF1-> Med-13. | X-3;  XI-2;  XII-2;  X-2;  XI-5;  XII-4. | pTAJAK-131;  pTAJAK-132;  pTAJAK-133;  pTAJAK-205;  pTAJAK-227;  pTAJAK-228;  pTAJAK-225. |

Supplementary Table 3. **Plasmid table.**

| Name | Parental plasmid | Features |
| --- | --- | --- |
| pTAJAK-122 | pCfB3036 | tADH1-ActI-1 <-pTDH3-pTEF1-> ActI-3-tCYC1 |
| pTAJAK-123 | pCfB3038 | tADH1-ActI-2 <-pTDH3-pTEF1-> SFP-tCYC1 |
| pTAJAK-131 | pCfB3034 | tADH1-ActIII <-pTDH3-pTEF1-> ActIV-tCYC1 |
| pTAJAK-132 | pCfB2903 | tADH1-ActVI-1 <-pTDH3-pTEF1-> ActVI-3-tCYC1 |
| pTAJAK-133 | pCfB3039 | tADH1-ActVII <-pTDH3-pTEF1-> ActVI-A-tCYC1 |
| pTAJAK-134 | pCfB2899 | tADH1 ActVI-4 <-pTDH3-pTEF1-> ActVA-6-tCYC1 |
| pTAJAK-135 | pCfB3037 | pTEF1-> ActVI-2-tCYC1 |
| pTAJAK-136 | pCfB3040 | tADH1-ActVA-5 <-pTDH3-pTEF1-> ActVB-tCYC1 |
| pTAJAK-204 | pCfB2904 | pTDH3-> AaOKS-tCYC1 |
| pTAJAK-205 | pESC-URA | pTDH3-> AaOKS-tCYC1 |
| pTAJAK-220 | pCfB3036 | tADH1-ActVI-2 <-pTDH3-pTEF1-> ActVA-6-tCYC1 |
| pTAJAK-221 | pCfB3038 | tADH1-ActVB <-pTDH3-pTEF1-> ActVI-3-tCYC1 |
| pTAJAK-225 | pCfB3040 | tADH1-Med-7 <-pTDH3-pTEF1-> Med-13-tCYC1 |
| pTAJAK-226 | pCfB3037 | tADH1-ActVI-2<-pTDH3-pTEF1-> ActVA-4-tCYC1 |
| pTAJAK-227 | pCfB2899 | tADH1-Med-29 <-pTDH3-pTEF1-> ActVA-6-tCYC1 |
| pTAJAK-228 | pCfB3037 | tADH1-Med-9 <-pTDH3-pTEF1-> ActVA-4-tCYC1 |
| pTAJAK-229 | pCfB2909 | pTDH3-> ActVA-4-tCYC1 |
| pTAJAK-121 | pTAJAK-71 | gRNA expression plasmid, targeting sites: XI-1, XII-1 |
| pCfB3051 | pTAJAK-71 | gRNA expression plasmid, targeting sites: X-3, XI-2, XII-2 |
| pCfB3053 | pTAJAK-71 | gRNA expression plasmid, targeting sites: X-2, XI-5, XII-4 |
| pCfB2909 | pTAJAK-71 | gRNA expression plasmid, targeting sites: XII-5 |

Supplementary Table 4. **Primer table.**

| Name | Gene amplified | Sequence 5’->3’ |
| --- | --- | --- |
| TJOS-136F | ActI-1 | AGTGCAGGUATGCCACTAGATGCGGCCCC |
| TJOS-136R | ActI-1 | CGTGCGAUTCAAGCAGCAGCTCCCGCCG |
| TJOS-137F | ActI-3 | ATCTGTCAUATGGCTACATTATTGACTAC |
| TJOS-137R | ActI-3 | CACGCGAUTCAAGCAGCTTCAGCAAGTG |
| TJOS-138F | ActI-2 | AGTGCAGGUATGTCTGTTCTTATAACAGG |
| TJOS-138R | ActI-2 | CGTGCGAUTCAGGGTGTTGGTGCAAAAC |
| TJOS-139F | SFP | ATCTGTCAUATGAAGATTTACGGAATTTA |
| TJOS-139R | SFP | CACGCGAUTTATAAAAGCTCTTCGTACG |
| TJOS-140F | ActIII | AGTGCAGGUATGGCCACACAAGACAGTGA |
| TJOS-140R | ActIII | CGTGCGAUTCAGTAATTTCCTAATCCCC |
| TJOS-141F | ActIV | ATCTGTCAUATGACTGTCGAAGTCAGAGA |
| TJOS-141R | ActIV | CACGCGAUTCAAGCCAGGCAAGTTGGTA |
| TJOS-142F | ActVI-1 | AGTGCAGGUATGTCTACCGTGACAGTGAT |
| TJOS-142R | ActVI-1 | CGTGCGAUTTATTTTGTCTCTTCTTGGG |
| TJOS-143F | ActVI-3 | ATCTGTCAUATGACTTCATCATTGCATCA |
| TJOS-143R | ActVI-3 | CACGCGAUTCACTTATGTTTTTTAACCT |
| TJOS-144F | ActVII | AGTGCAGGUATGAGCAGGCCAGGTGAGCA |
| TJOS-144R | ActVII | CGTGCGAUTTAACTAGCGGGTCCGGCTG |
| TJOS-145F | ActVI-A | ATCTGTCAUATGACAATTACAGCTCTGCC |
| TJOS-145R | ActVI-A | CACGCGAUTTAGGCGGGAAAGACATGGT |
| TJOS-146F | ActVI-4 | AGTGCAGGUATGCCCAAGGCAGTCGCTAT |
| TJOS-146R | ActVI-4 | CGTGCGAUTTAAAGGTCGGGAACCAAAA |
| TJOS-147F | ActVA-6 | ATCTGTCAUATGGCTGAGGTAAATGACCC |
| TJOS-147R | ActVA-6 | CACGCGAUTTAACTAGGCAAAATAGCCC |
| TJOS-148F | ActVA-5 | AGTGCAGGUATGTCTGAAGACACTATGAC |
| TJOS-148R | ActVA-5 | CGTGCGAUTTAGCCATCGTTTGACCTCC |
| TJOS-149F | ActVB | ATCTGTCAUATGGCGGCAGATCAAGGTAT |
| TJOS-149R | ActVB | CACGCGAUCTAGCCTGCGTGCGCAGGAA |
| TJOS-150F | ActVI-2 | ATCTGTCAUATGATGAGAGCAGTCCAATT |
| TJOS-150R | ActVI-2 | CACGCGAUTTAAGGAACTAAAACAACCC |
| TJOS-368F | AaOKS | ATCTGTCAUAAAACAATGAGTAGTTTATCAAATGC |
| TJOS-368R | AaOKS | CACGCGAUTCACATCAATGGCAAGGAAT |
| TJOS-372F | ActVI-2 | AGTGCAGGUATGATGAGAGCAGTCCAATT |
| TJOS-372R | ActVI-2 | CGTGCGAUTTAAGGAACTAAAACAACCC |
| TJOS-373F | ActVB | AGTGCAGGUATGGCGGCAGATCAAGGTAT |
| TJOS-373R | ActVB | CGTGCGAUCTAGCCTGCGTGCGCAGGAA |
| TJOS-375F | ActVA-4 | ATCTGTCAUATGCCGGATGAAAATAAGCC |
| TJOS-375R | ActVA-4 | CACGCGAUTTATGCAGGGTCCCTGGGAG |
| TJOS-376F | Med-7 | AGTGCAGGUATGCCTGCCACTCAGCCAAC |
| TJOS-376R | Med-7 | CGTGCGAUTTAAGGAGTATCCAAGGCTC |
| TJOS-377F | Med-29 | AGTGCAGGUATGTTATCTGTCGTGATGGA |
| TJOS-377R | Med-29 | CGTGCGAUTTATCTGTCTCCGTTGGTCG |
| TJOS-378F | Med-9 | AGTGCAGGUATGCCTTGTGCATTGTCTGA |
| TJOS-378R | Med-9 | CGTGCGAUTTAGGCGCCGCCTCCTGCAG |
| TJOS-381F | Med-13 | ATCTGTCAUATGACCGTGAGAGCCGACAT |
| TJOS-381R | Med-13 | CACGCGAUTTACGTCGCAGTACGGAACC |

Supplementary Table 5. **1H-NMR shifts and correlations found from DQF-COSY, ed-HSQC, H2BC and HMBC.**

| Position | 13C ppm | 1H ppm | integral | splitting | J (Hz) | COSY | H2BC | HMBC |
| --- | --- | --- | --- | --- | --- | --- | --- | --- |
| 1 | Not identified | OH not in spectra | - | - | - | - | - | - |
| 2 | 119.2 | - | - | - | - | - | - | - |
| 3 | 150.47 | - | - | - | - | - | - | - |
| 4 | 20.94 (CH/CH3) | 3.85 | [3H] | s | - | - | - | C2, C3, C5 |
| 5 | 124.48 | - | - | - | - | - | - | - |
| 6 | Not identified | - | - | - | - | - | - | - |
| 7 | Not identified | - | - | - | - | - | - | - |
| 8 | 161.9 | OH not in spectra | - | - | - | - | - | - |
| 9 | 124.72 (CH/CH3) | 7.30 | [1H] | d | 8.24 | H10 | C10 | C10, C8 |
| 10 | 118.26  (CH/CH3) | 7.67 | [1H] | t (dd) | 7.84 | H9, H11 | C9, C11 | C8, C12 |
| 11 | 135.69  (CH/CH3) | 7.60 | [1H] | d | 7.45 | H10 | C10 | C10, C9, C13 |
| 12 | 133.0 | - | - | - | - | - | - | - |
| 13 | 183.5 | - | - | - | - | - | - | - |
| 14 | Not identified | - | - | - | - | - | - | - |
| 15 | 115.95 (CH/CH3) | 7.23 | [1H] | s | - | - | - | C2, C13 |
| 16 | Not identified | OH not in spectra | - | - | - | - | - | - |

**Supplementary references**

[1] [Derksen GCH, Niederländer HAG, van Beek TA. Analysis of anthraquinones in Rubia tinctorum L. by liquid chromatography coupled with diode-array UV and mass spectrometric detection. J Chromatogr A 2002;978:119–27.](http://paperpile.com/b/7x3jzt/NtSW)

**Gene sequences used in this study:**

ActI-1

ATGCCACTAGATGCGGCCCCTGTTGACCCTGCATCTCGTGGTCCAGTGAGTGCCTTCGAACCCCCTTCAAGTCATGGTGCTGATGATGATGACGACCACCGTACGAACGCCTCTAAAGAATTGTTTGGTTTAAAAAGAAGAGTCGTAATTACAGGTGTAGGAGTACGTGCTCCCGGCGGCAATGGTACGAGACAGTTCTGGGAACTATTGACCTCAGGGAGAACCGCAACCAGGAGAATTTCCTTCTTCGATCCTTCACCGTACCGTTCTCAAGTAGCTGCAGAAGCGGATTTTGACCCAGTAGCTGAAGGGTTCGGCCCTAGGGAATTGGACCGTATGGATAGAGCATCACAATTTGCTGTAGCTTGCGCACGTGAAGCATTTGCAGCCTCAGGCCTAGATCCCGATACATTAGATCCAGCCAGGGTCGGAGTCTCTTTAGGAAGTGCGGTCGCTGCGGCCACAAGTTTGGAAAGAGAATATTTATTGTTGAGTGATTCAGGTCGTGACTGGGAGGTTGATGCTGCATGGTTAAGTAGGCACATGTTTGACTACCTTGTTCCGTCTGTTATGCCCGCGGAAGTTGCGTGGGCCGTCGGAGCTGAAGGTCCAGTTACTATGGTTTCAACAGGTTGTACGAGTGGTTTAGACAGCGTGGGTAATGCCGTCAGAGCAATAGAAGAAGGTTCAGCCGATGTTATGTTCGCTGGAGCAGCCGATACGCCAATAACTCCAATTGTGGTCGCATGTTTTGATGCTATCAGGGCTACTACTGCGAGGAATGATGATCCTGAACATGCCTCCCGTCCATTCGATGGAACAAGGGATGGCTTTGTACTGGCCGAAGGTGCTGCTATGTTTGTACTAGAGGATTATGACTCTGCGTTAGCTAGAGGTGCTAGGATTCATGCCGAGATTTCTGGATACGCAACTAGGTGTAACGCATACCATATGACAGGCCTAAAGGCCGATGGGCGTGAAATGGCTGAAACCATAAGAGTTGCTCTGGACGAATCCAGAACAGATGCCACTGATATCGATTATATAAATGCTCACGGTTCTGGGACACGTCAAAATGATAGACACGAAACTGCCGCATATAAGCGTGCATTGGGAGAACATGCCAGGAGAACCCCGGTTAGCTCTATTAAGAGTATGGTTGGGCACTCTTTGGGTGCGATTGGAAGTTTGGAAATAGCAGCCTGCGTGTTGGCTTTAGAACACGGTGTGGTCCCACCCACAGCCAATTTGAGGACTAGCGACCCTGAATGTGACTTGGACTACGTGCCCTTGGAGGCAAGAGAAAGAAAGCTGAGGTCCGTTCTAACCGTTGGTTCTGGATTTGGGGGGTTCCAAAGTGCTATGGTCTTGAGAGATGCTGAAACGGCGGGAGCTGCTGCTTGA

ActI-2

ATGTCTGTTCTTATAACAGGCGTTGGCGTTGTAGCACCTAACGGTCTGGGACTTGCACCTTATTGGAGTGCTGTTCTGGATGGCCGTCACGGCTTGGGCCCCGTTACCAGATTTGATGTGTCAAGATATCCTGCCACCCTTGCCGGTCAAATCGATGATTTTCATGCTCCAGACCATATCCCAGGAAGATTGCTACCTCAGACTGATCCGAGCACTAGGTTAGCGTTGACCGCCGCCGATTGGGCCCTGCAAGATGCGAAGGCTGATCCAGAAAGTTTGACGGATTACGATATGGGTGTGGTGACCGCCAATGCCTGTGGTGGGTTTGATTTTACTCATCGTGAATTTAGAAAACTTTGGTCAGAGGGTCCAAAATCAGTTTCCGTTTACGAATCCTTTGCATGGTTTTATGCTGTCAATACGGGTCAGATTAGCATCAGACATGGTATGAGAGGACCATCCAGTGCGTTAGTGGCTGAACAAGCTGGTGGACTTGATGCGTTAGGTCACGCTAGACGTACTATTAGAAGAGGGACACCATTAGTCGTGTCTGGAGGTGTCGATAGTGCTTTGGATCCGTGGGGTTGGGTTAGCCAAATCGCTAGCGGTAGAATTAGCACCGCCACAGACCCGGACAGGGCTTATCTTCCATTCGATGAACGTGCGGCAGGTTATGTTCCCGGCGAAGGTGGAGCTATATTAGTTTTGGAAGATTCCGCAGCTGCAGAAGCTCGTGGCAGGCATGACGCTTATGGAGAGTTAGCCGGTTGCGCATCTACCTTTGACCCTGCTCCTGGCTCCGGCAGACCAGCAGGTCTTGAAAGAGCTATAAGATTGGCTTTGAACGATGCGGGCACGGGACCCGAAGATGTAGATGTCGTTTTTGCTGATGGCGCAGGTGTACCAGAATTAGATGCCGCTGAGGCCAGGGCTATTGGTAGAGTTTTCGGAAGAGAGGGTGTACCTGTCACGGTTCCCAAAACTACTACAGGCAGATTGTATAGTGGTGGTGGGCCTTTAGATGTGGTTACGGCTCTGATGAGCTTAAGAGAAGGCGTCATTGCTCCAACAGCAGGAGTTACATCAGTTCCAAGGGAATATGGAATTGATTTGGTTTTAGGTGAGCCTCGTTCTACAGCTCCAAGGACTGCGTTAGTTTTGGCTAGGGGCAGATGGGGTTTTAATTCTGCCGCTGTCCTGAGGCGTTTTGCACCAACACCCTGA

ActI-3

ATGGCTACATTATTGACTACAGATGACCTGAGAAGAGCCCTTGTCGAATGTGCTGGTGAGACTGATGGCACAGACTTATCTGGCGACTTCTTGGATTTACGTTTTGAAGATATTGGATACGATAGTTTAGCTCTTATGGAAACAGCAGCGCGTTTGGAAAGCAGGTACGGCGTAAGTATACCTGACGATGTGGCAGGGAGAGTTGATACTCCGCGTGAACTGCTAGATTTAATTAACGGCGCACTTGCTGAAGCTGCTTGA

ActIII

ATGGCCACACAAGACAGTGAAGTGGCACTAGTTACTGGTGCAACTTCCGGAATCGGCTTGGAAATAGCAAGAAGACTAGGCAAAGAAGGGTTACGTGTATTTGTCTGCGCCAGGGGTGAGGAGGGACTAAGAACTACACTTAAGGAGTTAAGGGAAGCTGGGGTGGAAGCTGATGGAAGAACTTGCGACGTCAGATCTGTACCTGAAATAGAGGCTTTGGTGGCTGCCGTTGTCGAAAGGTACGGTCCAGTGGATGTCCTGGTTAACAATGCCGGAAGACCTGGTGGCGGCGCGACGGCTGAATTAGCTGACGAATTATGGTTAGATGTCGTCGAGACAAATTTAACAGGTGTGTTTCGTGTCACGAAGCAGGTGTTAAAAGCAGGAGGAATGCTAGAAAGAGGCACAGGGAGGATCGTAAATATTGCTAGTACAGGAGGAAAACAAGGTGTAGTACATGCAGCCCCTTATTCTGCCTCTAAACATGGTGTTGTTGGTTTTACAAAAGCATTGGGTTTGGAATTAGCTAGAACAGGTATTACTGTTAATGCTGTTTGCCCAGGCTTTGTTGAAACTCCGATGGCCGCCTCTGTCAGGGAGCATTACAGTGATATCTGGGAAGTTTCTACTGAGGAAGCATTTGATAGAATCACAGCTAGAGTCCCAATCGGAAGATACGTCCAGCCTTCAGAAGTCGCTGAAATGGTTGCATATTTGATTGGTCCAGGAGCTGCTGCAGTTACGGCTCAAGCACTTAATGTTTGTGGGGGATTAGGAAATTACTGA

ActIV

ATGACTGTCGAAGTCAGAGAAGTTGCAGAGGGGGTTTACGCCTACGAGCAAGCTCCTGGAGGTTGGTGCGTGTCCAACGCAGGGATTGTAGTCGGGGGTGATGGAGCTTTGGTTGTTGATACTCTTTCTACTATCCCAAGAGCTAGGAGATTGGCAGAGTGGGTCGATAAATTGGCCGCTGGACCTGGTAGAACCGTCGTCAATACTCATTTCCATGGTGACCATGCCTTTGGAAATCAGGTGTTTGCTCCAGGAACTAGAATTATCGCTCACGAAGACATGCGTTCCGCTATGGTAACAACCGGATTAGCACTAACAGGTTTATGGCCAAGAGTTGACTGGGGTGAGATAGAATTAAGACCACCTAATGTGACGTTTAGGGATAGACTGACACTGCATGTTGGTGAAAGACAAGTTGAATTGATTTGTGTGGGCCCTGCACACACAGATCATGATGTTGTAGTTTGGTTGCCCGAAGAAAGAGTTCTATTCGCCGGGGATGTTGTGATGAGTGGGGTCACTCCATTTGCGCTGTTCGGCTCAGTGGCTGGTACCTTGGCAGCCTTAGATAGATTGGCTGAATTAGAACCTGAAGTCGTTGTGGGTGGGCATGGACCTGTGGCCGGCCCCGAGGTTATCGACGCTAATAGGGACTACTTAAGATGGGTTCAAAGATTGGCAGCCGATGCTGTAGATAGGAGACTGACTCCACTTCAAGCAGCTAGAAGAGCTGACCTAGGCGCATTTGCTGGTTTATTGGATGCTGAAAGGTTAGTCGCTAACTTGCACCGTGCTCATGAAGAGCTATTGGGTGGTCATGTTAGAGACGCTATGGCTATTTTTGCGGAATTGGTGGCTTATAATGGTGGTCAATTACCAACTTGCCTGGCTTGA

ActVA-4

ATGCCGGATGAAAATAAGCCTGTATTAGTTCTAGGTGCCACGGGCAAGCAGGGAGGGAGTGCTGCTAGGTATCTTCTGGAAAGAGGCTGGACTGTTCGTGCTTTTGTTAGAGATCCAGGCGCTCCGAAGGCCAAAGAATTGAGAGAATTAGGGGCAAGTCTTCATACTGGTGATCTTGAAGATGCTGGTTCAGTTAGGGCAGCCATGAAGGGGGCATATGGTGTATTTTCAATCCAAACCCCGATGACGCCAGCTGGCGTCGAAGGGGAGGAGCGTCAGGGTAAGATTTGCGCAGACGCCGCCAGGGATCTGGGCGTCCAACACTATGTGCACTCATCTGTGGGAGGGGCTGAAAGACCTGAAGGAGTAAACTGGCGTCTTTCAAAGTTAGCCATCGAACAAAGAATTCAGGAAAACGCGCTACGTTTCACTTTCCTTCGTCCCAGTTACTTTATGGAAAATCTTAATCATGATATGAGTCCCCTGGTGATGGAAGATGGGGTACTGACTTTCAGAAGGGGTCTGGGACCTGCAAACACGCTGCAAATGATTTCAGGGCCTGATATCGGCTATTTCGCGGCGGATGCCTTCGATGACCCGGACACTTTCGGCGGCGCGAAAATTGAATTAGCTGGCGATGAACTTACCGGAGAGCAAATTGCCGCGGCGTTTGGTAGGCATACCGGGTTACCCGCTAGGTTTGTTAGCGTTCCGATACCTGAGTTACACAGGACCGGATTTGAGTGGCAGGCTATCAGCTACACTTGGTTGAACGGCATTGGCTACCATGCTGACATCCCAACTTTGCGTGCAAGGTTTCCGCAACTATTAACCCTAGACCAATGGTTAGCAAGAACCGGATGGACTCCCAGGGACCCTGCATAA

ActVA-5

ATGTCTGAAGACACTATGACCCAAGAACGTCCTTCCTTGACAGCTCACGCTCGTAGAATCGCTGAACTGGCTGGTAAGAGAGCTGCGGATGCCGAGCAACAAAGAAGATTGTCTCCGGATGTGGTTGACGCTGTGTTGAGAGCTGGTTTTGCTGCACATTTTGTCCCGGTTGCACACGGTGGTAGAGCTGCAACGTTCGGGGAATTAGTGGAGCCTGTCGCGGTTCTAGGGGAAGCGTGCGCTTCAACCGCCTGGTATGCAAGCCTGACAGCATCATTGGGTCGTATGGCAGCATATTTGCCTGACGAAGGTCAAGCGGAATTGTGGAGCGACGGTCCAGATGCGCTTATCGTGGGAGCATTAATGCCGTTAGGTAGGGCTGAAAAAACGCCGGGTGGCTGGCATGTCTCAGGTACTTGGCCCTTTGTCTCAGTGGTTGACCATTCCGATTGGGCATTAATCTGTGCTAAGGTAGGGGAGGAACCCTGGTTTTTTGCCGTACCTCGTCAGGAATATGGGATTGTCGACTCTTGGTATCCCATGGGTATGAGAGGGACCGGTTCAAATACTCTTGTGTTGGATGGCGTTTTCGTACCTGATGCCAGAGCATGTACAAGAGCGGCCATCGCTGCAGGTCTAGGCCCAGACGCAGAAGCCATCTGTCACACCGTTCCAATGAGAGCTGTCAACGGACTAGCGTTTGCTTTACCTATGCTTGGTGCCGCTAGGGGTGCAGCTGCTGTGTGGACGTCATGGACTGCAGGGCGTTTAGCTGGTCCAACTGGTCAAAACGCTGTTAGCTCCCAAGATCGTGTGGTCTACGAGCACACACTGGCAAGAGCTACTGGTGAAATTGATGCTGCTCAGTTATTGCTAGAGAGAGTCGCCGCCGTTGCTGACGCCGGATCAGCTACAGGTGTTTTAGTGGGTAGAGGTGCAAGAGATTGTGCTTTGGCGGCCGAGCTTCTTACAGCGGCAACTGATAGACTTTTTGCATCTGCTGGTACCAGGGCTCAAGCTCAAGACTCCCCAATGCAGAGGCTATGGCGTGACGTTCATGCAGCAGGGTCCCATATTGGGCTACAATTTGGTCCAGGCGCCGCACTTTATGCTGGTGAGTTATTGAGGAGGTCAAACGATGGCTAA

ActVA-6

ATGGCTGAGGTAAATGACCCAAGAGTCGGGTTTGTTGCAGTTGTCACATTTCCAGTTGATGGTCCTGCAACACAACACAAGCTAGTGGAATTAGCTACAGGCGGTGTTCAAGAATGGATTCGTGAAGTTCCTGGCTTCCTTAGTGCTACATACCATGCATCAACTGATGGCACAGCTGTTGTTAATTATGCACAATGGGAAAGTGAACAGGCTTATAGAGTTAATTTCGGCGCTGACCCAAGATCTGCTGAACTTAGAGAAGCATTATCTTCCTTACCTGGTCTGATGGGCCCACCAAAAGCCGTTTTCATGACCCCAAGAGGGGCTATTTTGCCTAGTTAA

ActVB

ATGGCGGCAGATCAAGGTATGCTTCGTGATGCGATGGCCAGGGTCCCTGCAGGTGTAGCTCTGGTCACTGCACATGACCGTGGCGGTGTGCCGCATGGATTCACAGCTTCCTCATTCGTTTCTGTCAGTATGGAACCACCCTTAGCTTTGGTGTGTTTAGCCAGAACTGCTAATAGTTTCCCAGTATTTGACTCTTGTGGGGAATTTGCTGTTTCTGTACTGAGGGAAGATCATACCGATCTTGCCATGAGATTTGCAAGAAAGTCAGCAGATAAGTTCGCTGGTGGGGAGTTTGTTAGAACCGCAAGAGGAGCAACTGTCCTAGACGGTGCAGTTGCAGTAGTTGAATGTACTGTGCATGAACGTTACCCAGCCGGTGATCATATCATTTTACTGGGTGAGGTGCAATCCGTTCACGTAGAGGAAAAAGGTGTACCAGCTGTGTACGTAGATCGTAGATTTGCCGCGTTGTGCTCTGCTGCCGGAGCATGTCCGTCAGCAACCGGTAGGGGCGTTCCTGCGCACGCAGGCTAG

ActVI-1

ATGTCTACCGTGACAGTGATAGGTGCTGGCACCATAGGTTTGGGCTGGATCAATCTTTTCTCCGCAAGGGGCCTGACTGTTAGAGTTAACAGTAGAAGACCCGACGTCAGACGTGTAGTCCATGAAGCATTGGAATTATTTTCACCTGGTAGGGTCGATGAATTAGCAGCTAGAATTGAATATGAACCAGATGTTGGGAGAGCTGTAGCAGGCGCTGATGTTGTCTCTGAAAATGCTCCGGATGATTTGCCTTTAAAGCAACGTTTGTTCGCTGAAATTGGTGCAGCTGCTCCAGATCATGCCTTAGTTCTTTCTTCAACGAGTAAACTGCTACCTGATGAACTTTCTCGTGATATGCCTGGACCCGGGAGACTGGTTGTGGCTCATCCATTTAACCCTCCCCACATTGTTCCATTAGTGGAGGTTGTTAGAGGCGAAAGAACCGACCCGGAAGCCGTTGAAAGAACGCTAGCTTTTTTAGCGTCTGTGGGGAGAACGCCAGTTGTCGTTAGAAGGGCTTTGCCAGGGTTTGCTGCAAACCGTTTACAATCAGCATTGCTAAGAGAATCAATTCATTTAGTCTTAGAAGGTGTGGTTACAGTTGAGGAACTAGATAGAATTGTGACCGATTCCATCGGGCTAAGATGGTCTACTATCGGTCCGTTTCATGCTTTTCATTTGGGGGGTGGTCCAGGTGGCTTGAGAAAATGGTTGGAACACCTTGGCTCAGGTCTTGAACAAGGCTGGAGAGGGCTTGGGCAGCCAGCTTTAACACCACAAGCGGTTGAAGCTTTGGTTGCTCAAACTGAAGCAGCGTACGGGCACAGACCTTATGCTGAGTTAGTTAGGGATAGAGATGACAGACACTTGGCGGTACTAGCAGCGTTAGAAAGAACTGAGCAACCCCAAGAAGAGACAAAATAA

ActVI-2

ATGATGAGAGCAGTCCAATTCGATAGGTATGGGGACCCAGACGTTCTGTATGTTGCCGAAAGGCCAGTTCCCGAACCAGGGCCTGGTCAAGTTAGAATCGCAGTCGATGCTGTTTCCGTGGGTCACGCTCAGACACAGATGAGAAGGGAAGCATTTCCTGCTCCGATGTGGCGTCCAGTATTTCCGGTGGTATTAGGTGGCGATGTAGTTGGTAGGATCACAGCTGTAGGACCAGGCGTTACAGCACTAAGACCAGGGGACAGAGTCGGTGCTTTTACTCTGTATGGTGCATATGCAGAACAAGTCGTGGTTGACGCCGTCACTGTTGTCCCAGTCCCCGAAGAATTAGATGCTGCCGAGGCCGCTGTTTTACCTGGTACTGGACTTATCGCCTTAGGTATACTAAGGACGGGACGTTTGAGAAAGGGAGAGACAGTTTTAATTCATGCGGCAGCTGGTGGTGTAGGTCATATAGCGGTCCAATTAGCTAGAGCAGCCGGTGCTGGTTTAATTATCGGAACGGCAGGGGCTGCGGCGAAAAGAGAGTTCGCGAGAGCAACTGGTGCAGACGCAGTTGTGGATCATAGAAGTGCACACTGGGCAGACGAAGTCCGTGAATTAACTGGAGGACGTGGTCCAGATCTTATCCTGGATGGCATTGGCGCAGAAGTCCTTGAACAAGGTGTAGGCTTGTTAGCTCCTGGCGGGAGACTGGTTTTTTATGGTAGTTCTGGTGGGGAACTAGCGATTCCTAAAGTATCCGTGATGGACTTAATCGGAATTAAATATGTTACTGGTTTCGCTCTAAGTGCTTGGAGAGGTGGAAGACCTCAAGAGTACGAAGCTGGGGTGGCTGAATTAACTAGACTTCTGGCTGAGGGTCAAGTTACGTCCGCAGTGCATGCCAGATTACCCCTAGAAAGAGCTGCTGAAGCCCATGCTGTCGTAGAAGCTAGAGCTCAATTAGGCAGGGTTGTTTTAGTTCCTTAA

ActVI-3

ATGACTTCATCATTGCATCACGCTATCAGACTAACAACCGCGTCAGCTATAGCTTTAGGCGGGTTAGTAACCTTGGGCACGTCCGCACATGCAGCAAGTGTTGCAGTTCCATACGAGTGCAGAACCTGGGTCCAGGGTAATACGCATCCTGTTTACGACTATGCAAGGGGGTTTGATGTAAGCGTCCCCGCTTCTGTTAGAGCTGGTAAAAAATTTAAAGCAACCTACGATCCTGATCCAATCACAGCTTTCGCCGAATACAATCAGATCGTTAACGATGTTAGAATAGCTTACAGAATACCGGATGGTGCTAAGGTTCATAAGGTTAGGCTGACTGGGGGAAGCGGCTTAGGGGATTCTGACGTAAGAGTTCAAGTTAAAGGAAGAGATATTGTCGTGTCAGCCAGCGGCCCATTTCAAGGTGGCGTAGAGTTTGATTTACCCACACTAAAAGTTACATACAAAGCGCCCAAGACAACTGGTCCATTGAACTTTGTTAGTGGCGGTTCTGGATACGAAGATCCTGGTTTCTACTGGTATCGTTACCAACCTATTCTTGACGAGTGGGGACCATTTGAATGCTTTCCAGACCCTGCTAAACCAGAAGCTGTGCTTGCCTCTACCCAGGTTAAAAAACATAAGTGA

ActVI-4

ATGCCCAAGGCAGTCGCTATTCATCAATTTGGTGGTCCTGATGTATTAGGATTGGTCGATGTACCGGAACCTGTGCCTGGCCCTGGTCAAGTTACCGTGAGAGTCAGAGCTGCTGGCGTCAATGGCTTTGACTGTCGTGTTAGATCTGGTGGAATGAGAGGGAGATATCCAGTTGAATTTCCCCAAATAATCGGAAATGAATTTGCCGGTGTAGTCGAAAGAACTGGTCCGGAAGCGACCGGGTTTGCTCCAGGTGACGAAGTCTTAGGCTTTGCAGTCTTGCAAAGTGGTACGGAGCTGCTAGCAGTCGGAGCTGACCAAATAACTGTCAAACCACCGGAGCTTAGTTGGGAAGTGGCTGGGAGTTTATCAGCCGTAGGCCAAACTGCAGACATAGCCCTTGCAGAACTAAGAGTTGGGCCTGGTGATACAGTACTGGTTCACGCTGCTGCAGGGGGGGTCGGTAGCTTAGCTGTCCAATTAGTTCGTGAAAGGGGTGGGACTGCAATCGGGTCAGCAGGAGAACACAATCATGATTATTTACGTTCTCTGGGTGCCTTACCTGTTGCTTACGGTCCCGGATTTGCAGACAGAGTGCGTGCATTGGCACCAAACGGCGTGGACGCAGCTCTAGACTGTCATGGAGGTCCTGAAGCCTTAGCTGTTTCTTTGGAACTGGTGGCCGATCGTGCGAGAATTGCTACTGTTGCAAATTTCAGAGCTGCCGCACAAGAAGGTATTGTGATGCCTAGGGTAGTGAGATCTGCCGAAAGGTTGGCCGCGCTTGCAGCCCTTTGTGCCGAAGGCAGATTAAGACCTCATGTCGAAGCAGTTCTGCCCTTTGCCAAAGCTGCCGAAGCACACCATCGTCTGGAACAAGGTCACGTGAGAGGGAAACTAGTTTTGGTTCCCGACCTTTAA

ActVII

ATGAGCAGGCCAGGTGAGCATAGGGTAGTTCATACGTTAAGAACGCAAGCTCCAGCCAGGAGATTGTATGAGTTAGTTGCTAGAGTTGAAGATTGGCCCGCTGTTTTCGAACCTACTGTTCATGTACAAGTGTTAGAGCGTGGTCCAGGGACCGAAAGGTTCAGAATCTGGGCAAGAGTGGGGGGAAGAGTCAAAACGTGGACATCACGTAGAACACTTGATCCCGATACACTACGTGTTACCTTCAGGCAAGAATTGACACAACCCCCTATCGCTTCCATGGGTGGTAGCTGGGAATTTAGAGGTGACGGAGATGGTACAGAAGTTGTTCTAACACACGACTTTGCTGCTGTTGATGAAGCGGCGCTGCCAGGACTTAGAGAGGCTCTAGACGCTAATTCTGGAAAGGAACTAGCGGCATTGGTGGCCTTAGCTGAAAGACGTCAACCTCCTGAAGAATTGGTATTTACCTTTGAAGATACGCTTAGAGTTCCATCTGGAGACGATGCGTATGCTTTTATCGAAAGAAGTGATTTATGGCAGGAAAGGCTTCCACATGTAAGGAAAGTTACTCTAACCGAAGAAGCAGCAGGCACAGGTCCGGCAGAAACTAGAGATATGACAGTTCAGGACATGACTATGGAAACAGTGACGACCGATGGCGGTACCCATACAACTAGGAGTATCAGACTGTGCGTTCCGGCTAGGTCAATCGTCTATAAGCAATTGGTCCCACCAGCACTGTTATCAGGTCACTGTGGCGCATGGACATTTGGTGAGGATACTGTTACAGCTAGACACACAGTTGCTATTGATCCTGCAAGGGTTGAAGAAGTGTTGGGTAAAGGAGCTACGGTGGCGGACGCTAGAACTCATCTTAGAGAGGTGTTGGGCGCTAATTCCAGAGCTACCCTAAGACATGCTGCTGCGGCAGCCGGACCCGCTAGTTAA

SFP

ATGAAGATTTACGGAATTTATATGGACCGCCCGCTTTCACAGGAAGAAAATGAACGGTTCATGACTTTCATATCACCTGAAAAACGGGAGAAATGCCGGAGATTTTATCATAAAGAAGATGCTCACCGCACCCTGCTGGGAGATGTGCTCGTTCGCTCAGTCATAAGCAGGCAGTATCAGTTGGACAAATCCGATATCCGCTTTAGCACGCAGGAATACGGGAAGCCGTGCATCCCTGATCTTCCCGACGCTCATTTCAACATTTCTCACTCCGGCCGCTGGGTCATTGGTGCGTTTGATTCACAGCCGATCGGCATAGATATCGAAAAAACGAAACCGATCAGCCTTGAGATCGCCAAGCGCTTCTTTTCAAAAACAGAGTACAGCGACCTTTTAGCAAAAGACAAGGACGAGCAGACAGACTATTTTTATCATCTATGGTCAATGAAAGAAAGCTTTATCAAACAGGAAGGCAAAGGCTTATCGCTTCCGCTTGATTCCTTTTCAGTGCGCCTGCATCAGGACGGACAAGTATCCATTGAGCTTCCGGACAGCCATTCCCCATGCTATATCAAAACGTATGAGGTCGATCCCGGCTACAAAATGGCTGTATGCGCCGCACACCCTGATTTCCCCGAGGATATCACAATGGTCTCGTACGAAGAGCTTTTATAA

ActVI-A

ATGACAATTACAGCTCTGCCCACGGGGTTGTATGCCGAAGTGTTAAGCTTTTATGGACATCAAATGCAGAAATTAGATGGCAGAGATTTTGCTGGATACGCTGCAACTTTCACTGAAGATGGCGAATTTCGTCATTCACCATCATTGCCTGCTGCCCATACACGTGCAGGGATAACAGCCGTACTTGAAGATTTCCATAGAAAGTTCGATGCTCGTAAGATACAAAGAAGGCATTGGTTTGATCATACAGCATTATCCCAGGCTTCTGATGGGTCAATAACCGCTACTTCCTATTGTTTGGTACTGACGGTCCACGCCGATGTCAAGGCTCCTGAATTCGGCCCAAGCTGCTTAGTTCACGACGTTTTAGTACGTGGTGCTGATGGTGAACTATTGTTAAGGTCCCGTCACGTAACCCACGACCATGTCTTTCCCGCCTAA

AaOKS

ATGAGTTCACTCTCCAACGCTTCCCATCTGATGGAGGATGTGCAGGGCATCCGGAAGGCCCAGAGAGCCGATGGCACGGCCACCGTCATGGCCATCGGAACAGCTCACCCTCCTCATATCTTTCCTCAGGACACCTACGCTGACTTCTACTTCCGCGCCACCAACAGCGAGCACAAGGTCGAGCTCAAGAAGAAGTTCGATCGCATCTGCAAAAAGACAATGATAGGCAAGCGCTACTTCAACTACGACGAGGAGTTCTTGAAGAAATATCCCAATATCACTTCATTCGATGAGCCCAGCCTCAACGACCGCCAGGACATTTGTGTCCCTGGGGTGCCAGCCCTGGGAGCCGAAGCAGCTGTGAAAGCCATCGCGGAATGGGGACGCCCCAAGTCTGAGATTACTCATCTCGTGTTCTGCACCTCCTGCGGTGTCGACATGCCCAGCGCCGACTTCCAGTGCGCCAAGCTCCTTGGCCTCCGCACCAATGTCAACAAGTACTGCGTCTACATGCAAGGATGCTATGCTGGTGGCACCGTCATGCGGTATGCCAAGGATCTGGCCGAGAACAACCGTGGTGCTCGTGTTTTGGTGGTGTGTGCGGAGCTCACCATAATCGGGCTTCGAGGCCCTAATGAGTCCCATCTCGACAACGCCATCGGAAATTCTCTTTTCGGAGATGGAGCTGCCGCGTTGATCGTCGGGTCGGACCCCATCATCGGTGTCGAGAAGCCCATGTTCGAGATCGTGTGTGCCAAGCAAACTGTGATCCCAAACAGCGAAGACGTTATCCATCTCCACATGAGAGAGGCAGGTCTGATGTTCTACATGAGCAAGGACAGTCCCGAGACCATCTCCAATAACGTAGAGGCTTGCCTCGTTGATGTGTTCAAGTCTGTGGGGATGACTCCTCCCGAGGACTGGAACTCTCTCTTCTGGATCCCTCACCCCGGTGGTCGCGCCATCCTTGATCAAGTTGAGGCCAAGCTGAAGCTTCGTCCTGAGAAGTTCCGTGCGACTCGAACCGTGCTCTGGGATTGCGGTAACATGGTCAGTGCGTGTGTGCTCTACATATTGGATGAGATGAGAAGAAAATCCGCTGATGAAGGACTAGAGACCTACGGAGAGGGACTAGAGTGGGGTGTCTTGCTTGGATTTGGACCAGGGATGACCGTTGAAACTATCCTTCTCCACAGCCTGCCTCTCATGTGA

Med-7

ATGCCTGCCACTCAGCCAACGAGTCTTTCACCCGATACAGTGAGAGAGGCTGCAGAGACGGCAGCTAAGCATGCAGCCGCATCAGAAGCTGAAAGGCGTTTGCCTCCGGAGGTTGTTGATCCTATACTGGCGGCAGGCTTTGCCAGACATTTTGTTCCAGCAAGGTGGGGAGGAAGCGCAGGCACCTTTGGCGCTCTGCTACCTGCAGTCACCACTATTGGAGAGAGTTGTACTAGCGCCGCTTGGTTTGCAAGTTTGGCAGCAAGTGTCGGCAGGTTAGCGGCCAACCTACCTACGGAAGGACAAGCAGAGCTATGGGCGGACGGGCCCGACACACCCTTGGTTGCGGCACTGGCGGCGGCCGGTACCGCGGAGAGAGTGCCTGGCGGGTGTCCGTCTGCACCTGTTCCTTATGTATCAGGAGTCGATAGCAGTGCATGGCCACTGGTATTTGCAAGAGCTCTGGATGGAGATCATCAGGAACCCAGATTTTTTGCTGTTCCACGTGCGGCATATGGTCTTGGCGATAGTTGGTTTAATGTTGGGCTAAGAGCCACGGGCAGCAATACTCTGGTTTTGGATGGGGTAACAGTCCCCGACACTCGTTCAGTGGCTTTAGCAGAGCTACTTGCAGGCAGAGCTCCTGAGGCGGAAGCACGTTGCCATCGTGTACCATTAAAGGCTGCGAACGGGCTAACACTAACTGCACCCCTTCTGGGAGCCGCGAGGGGCGCGTTACGTCATTGGTCTGGGTTGGTGCGTGGTAAGTTGCAGGCGCCGGCAGCTGCGGTAACGGGGGCAGCCGACAGAACTCCTCATGAATTGACCCTGGCTCGTACGGATGGCGAAGTAGATGCCGCCGCACTATTATTAGGGCGTGTTGCGGAAGTTCTTGACCACGATGCAGTTGACCCGCTACTGACCGCCAGGAACGGTCGTGATTGTGCCCTTGCTGCCGAATTACTGGCTGGCGCGGTCGATCGTTTGCTGAGGTCATCAGGGAGTCGTGGACAGGCCGACACCGAACCGTTGCAAAGATTCTGGCGTGATGTAAACGCTGGCGCCGGTCATTCCGGGCTACAATTTCCTCCGGTGGCCAGTGCGTTAGTCAGAGCGTCAAGAGCCTTGGATACTCCTTAA

Med-9

ATGCCTTGTGCATTGTCTGAAAGTATGTCAACTGCCGCCAGAATGGTGAGGACTGAGGAAGTCCCGATCCCGGAGCCTGGACCAGGTCAGGTGTTGATAGAAGCAGAGGCTATAAGCGTATTTGCTCAGACACAGATGAGGAGAAATGTATTCCCAGCACCCATGTGGAGACCTGAGTTCCCCATTACCTTAGGAGGTGATGTAGTAGGGGAGGTTGTTGCTGTAGGACCAGGCGTCACCGGGAATGCGCCTGCTACGGGCAGCGCTCCGTCACCGTGGCATGGGGCATACGCCGAATATGTTGTGGTCGATGCTGACACGGTCTTGCCCGTCCCCGCTGCTCTTGATGCTGCTCAAGCAACAGCACTACCGGCACCGGGACCAATTGCTATGGGAACAATGGGCACTGCGTCCTTAGCACCGGGAGAGTCCGTCCTAGTACAGAGACCTTCAGGAGGAATAGGTCACTTAGCAGTGCAATTAGCGAGACTGGCGGGTGCAGGTACCGTAATAGCCACGGCGGGAACTCCCGAGAAGTGCGCTTACGCGCGTAGCCTAGGTGCGGACGTAGCGGTAGACTACAGCAGAGCAGACTGGCCTGATGCAGTTCGTGAGGCCACAGGAGGTAGAGGTGTCGACGTTATTCTTGACTCAGTTGGAGGGGATGTCCTGCGTCAGGGTGTTGGTTTACTTGCGCCTTTTGGCAGGCTAGTTTTCTATGGTAGTGCCGGGGGCGGCCTGGGAATACCTGCAGTTAGTCCTATGGAGTTGATAGGGATGAGGTTTCTAACGGGATTTGCGTTATCCGCCTGGCGTAATGCGAGACCTGAGAGGTATAGAGCGGACCACGACGCGCTAACTGCTCATCTTCTAGCTGGTACGGTACGTCAAACGGTCCATGCCACCCTGCCGTTGGAAGACGTGGGTAAAGCTCATGAAATCGTTGAATCCCGTGCGCAGTTAGGGAGGATCGTTTTGCTTACAGGAAGGGGTACTAGAACTGCAGGAGGCGGCGCCTAA

Med-13

ATGACCGTGAGAGCCGACATTAGGTCCGTCAGAGACGCTGACACGTTCAAAGATGCTTTGTCTTTGCTTGCAAGTCCTTTGACTCTAGTGACGACTGCGGACGAGGAGGGACGTCCATGGGGATTTACTGCGTCTTCAGTCACGTCTGCTAGTCTGTCACCTCCATTAGTTATGGTCGGTATGGCGAGAACCTCCTCTTGCGCCGCCGCTCTTGACGCGGCGAGCGAGTTCGTAGTCAATGTGTTGGGTGAACAACACGAGGAGCTAGCCCGTCGTTTTGCGACTAGAGGAATAGATCGTTTCGCGGGACAGGGATTGGGGGCATGGCCTGGAACAAGACTGCCTTTCGTGCCCGATGCCCATGCTGCTTTCAGGTGCGCTGTTGAGGACAGATTAAGGGTCGGGGATCACGAACTGTTAGTGGGAAGACCAACAGAAGTGCTACTAGATGGAGGCTCTAGGCCTTTACTTTGGCATAGGCGTGGGTTCCGTACTGCGACGTAA

Med-29

ATGTTATCTGTCGTGATGGAGGAATTTGGAGGGCCGGAGGTCTTAAGGGCGAGACAGGTTGAGGACCCTGAGCCGGGTCCCGGGCAAGTACTTGTTGACGTGGCATATGCGAGCGTTACATTCGTGGAAACCCAAGTAAGATCAGGGAATGGACCGTTTGGTAGACCTGCGTTACCTAGGGTGCCGGGGAACGGCGTAGGTGGCAGAGTTGTAGCTTTGGGACCTGGCGCCGACCCCGCATTGTTAGGGACGGTCGTGGTGACCACCACTGGAGGCGAAGGAGGATACGCGGAGCGTGCACTAGCAAGAGCCGACGAAGTAGTACCGGTACCGCCGGGCTTGGCACTTAAGGATGCAGTGGCGCTACTGGCTGACGGTCGTACCGCTCTACTTCTGTTTAGACAGGCGGAAGTCAAACCAGGTGAAAAGGTTTTAGTCGAAGCTGCGGCCGGTGGCGTTGGTAGTTTGTTAGTTCAGCTTGCAGTTCACGCAGGGGCGCGTGTTGTTGGCGCCGCCAGAGGAGACAGGAAGGCGGAGTTAGTGGTCAGTTTAGGAGCGAGTTATGTAGACTACAGCAGGGAAGCCTGGCTAAGGCAAGTCCGTGAGACTGCAGGTGGCGGCGACTTAGATGTCGTATTTGATGGCGTTGGTGGAGCTATCGGTACCGCCGCAGCTGGCGCGCTTCGTCCCGGTGGAAGGATAAGTCTGTATGGAATGGCCTCAGGTGCGGACGCAACGCTTGATGAGGACGACTTGGCAGCCAGAGGTATACGTACACTTGGCTTGTTCGCCGCGCCAGGTCCGGCGGAGACCCACCCTTTAATAGGTGAGGCGCTGAGACTGGCGGCGGATGGCGTGTTGGCGCCTGTAGTAGGGCAAGTCTTTCCTTTAGCTGCCGCAGCCGAGGCACATGCGGCTATCGAGGCGCGTGCTACAGTAGGTAAGACCTTATTAGCGGTTGGTGCACAAGAAGGTGACACCTATGGTACTGGGGCGGCAGGCGGAGCGACCAACGGAGACAGATAA
